# Synthetic Lignin Oligomers: Analytical Techniques, Challenges, and Opportunities

**DOI:** 10.1002/cssc.202402334

**Published:** 2025-03-17

**Authors:** Myriam Rojas, Frederico G. Fonseca, Ursel Hornung, Axel Funke, Nicolaus Dahmen

**Affiliations:** ^1^ Scale-up of processes with renewable carbon sources Institute of Catalysis Research and Technology – Karlsruhe Institute of Technology (IKFT-KIT) Hermann-von-Helmholtz-Platz 1 76344 Eggenstein-Leopoldshafen Deutschland; ^2^ Simulation and Virtual Design Institute for Low-Carbon Industrial Processes – German Aerospace Agency (DLR) Walther-Pauer-Straße 5 03046 Cottbus Deutschland

**Keywords:** oligomers, lignin, spectroscopy, surrogates, biogenic platform chemicals

## Abstract

Lignin is the second most abundant renewable material after cellulose. However, its economic use is currently relegated to low‐value energy production. This biomaterial holds great potential as a source of renewable biofuels, bio‐based chemicals, advanced materials, and integrated biorefineries. Fractionation and depolymerization methods yield liquid repositories of promising aromatic monomers and lignin oligomers (LO) that retain many of the structural components found in the native material. However, analyzing this complex mixture is challenging due to the wide range of molecular sizes and heterogeneous chemical structure, which makes their structural elucidation a critical obstacle – unlocking the full potential of lignin hinges upon developing appropriate standards and analytical methods to address existing knowledge gaps. This review provides a comprehensive examination of current analytical techniques for elucidating the chemical structure of lignin oligomers, exploring synthesis methods, molecular structures, and their advantages and limitations. Built upon these findings, opportunities for synergy between synthetic oligomers and lignin utilization can be revealed, such as bioactive compound production and biorefinery integration. Moreover, we underscore the need for standardized analytical methods to facilitate the design of lignin oligomer standards and their diverse applications.

## Graphical abstract

The review begins by discussing lignin and its structural complexity, followed by challenges in elucidating depolymerization products and the analytical techniques used. It then focuses on the chemical structure of synthetic lignin oligomers, their synthesis mechanisms, and their significance in current science. Finally, scientific gaps are identified, and future research directions are proposed to guide scientists reading this work.

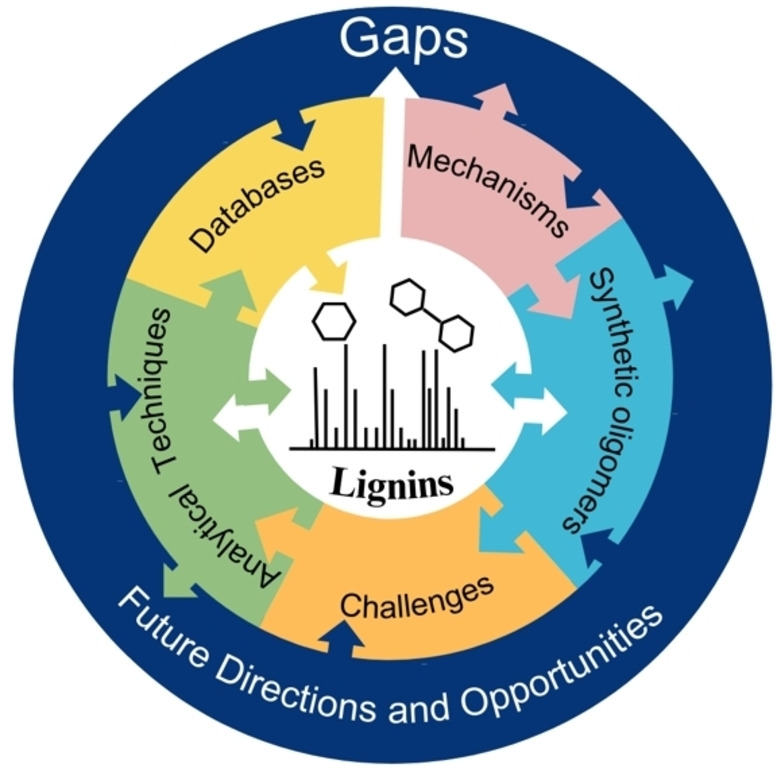

## 1 Introduction

The Earth′s surface temperature has increased since the late 19^th^ century, with a more rapid warming trend observed in recent decades. According to scientific records, the average global temperature has risen by 0.820 °C (land) and 0.437 °C (ocean) per decade since 1980. The 2010s were the warmest decade on record, with global temperature increases of 1.376 °C (land) and 0.651 °C (ocean).^[1]^ In 2023, the United Nations announced a new era of “global boiling,” highlighting the need for continued research and awareness about climate change.^[2]^

One of the main contributors to global warming is the emission of greenhouse gases, with the energy sector being the largest emitter at around 34 BtCO_2_e, followed by Land‐Use Change and Forestry at around 1.3 BtCO_2_e.^[1]^ Policies promoting the investment in renewable energy sources and the staggering diminution of costs of low‐scale technologies like solar and wind turbines[^3^, ^4^] have led to a reduction of over 30 % in CO_2_ emissions in Europe (EU27) in the period 1990–2021. More conservative politics addressing greenhouse gas emission reduction, such as those observed in the USA or Japan, led to diminutions of around 2 % and 8 %, respectively. However, large developing economies, such as Brazil, China, and India, present a relative increase of 124 %, 362 %, and 369 %, respectively, in the same period.^[5]^

Lignocellulosic biomass is a renewable and globally available resource that offers a short‐term carbon‐neutral alternative for producing hydrocarbons, direct heat, and power. Its share as an energy source has increased from 10.5 TWh to 15.4 TWh due to growing global energy demand.^[6]^ Biomass holds promise for developing liquid fuels, especially for hard‐to‐abate aviation and marine sectors.^[7]^ Biogenic side streams are preferred as input materials to ensure sustainability and avoid competition with food production.^[8]^

**Figure 1:**
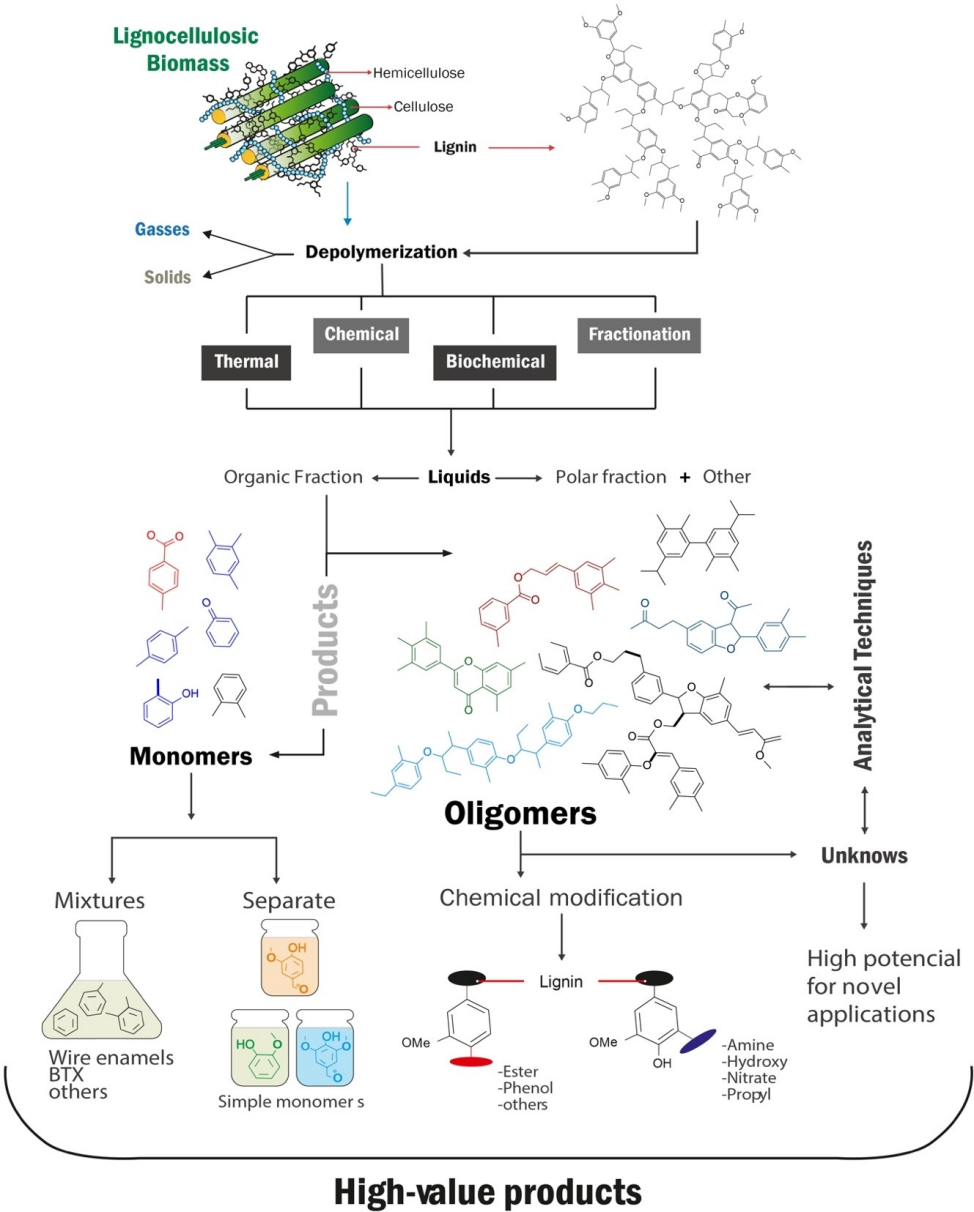
Lignin sources and biorefining process (lignocellulosic and thermochemical biorefinery).

Lignin in lignocellulosic biomass (Figure 1) is a residue from industries like cellulose, cellulose pulp, bioethanol, and emerging biotechnologies for value‐added chemicals/products. Despite being produced in large quantities, lignin is undervalued and often burned for process heat or discarded. However, its renewable and highly aromatic nature has generated growing interest in academia and industry.^[9]^ Unfortunately, there have been limited advancements in large‐scale commercial use of lignin.^[10]^ One of the biggest challenges today is the detailed structural elucidation of the total structure of native lignin, which is essential to understanding the various existing conversion pathways and generating new effective valorization pathways.^[11]^

This problem extends to all lignin obtained from lignocellulosic sources, e. g., lignin derived from sugarcane, as well as products derived from diverse depolymerization processes (see Figure 1): thermal, chemical, and biological, subsequently referred to as lignin oligomers (LO).^[12]^

Direct thermochemical liquefaction (DTL) is a process designed to maximize the production of liquid, biobased commodities. Two popular variants of DTL are fast pyrolysis (FP) and hydrothermal liquefaction (HTL), which target the production of fast pyrolysis bio‐oil (FPBO) or biocrude (BC), respectively. Other techniques that also produce significant quantities of lignin‐rich liquid products are catalytic hydrogenolysis/hydrosolvolysis, which takes place in organic solvent instead of water.[^13^, ^14^] Analyzing the complex mixture of molecules present in FPBO and BC, as well as the products obtained by their fractionation, is also a significant scientific obstacle. This difficulty arises because LO, the main weight‐wise component, features a wide range of molecular sizes and chemical properties.^[15]^

LO are intermediate molecular weight compounds derived from the depolymerization of lignin (detailed in section 1.3). These molecules consist of multiple linked phenolic units and are characterized by a variety of chemical bonds (detailed in section 1.1), including carbon‐carbon (C‐C) and carbon‐oxygen (C‐O) linkages. LO can be classified based on their size into monomers (single unit), dimers (two units), trimers (three units), tetramers (four units), and larger molecules containing even more linked units.^[16]^ Some LO can be recovered during thermochemical depolymerization, which applies thermal energy to disintegrate lignocellulosic feedstocks into liquid, gas, and solid products (Figure 1).

In the case of LO present in FPBO/BC, one of the main obstacles to achieving the structural identification of these chemical species lies in the absence of these species in commercial mass spectral libraries and the lack of specialized chemical standards explicitly designed for their analysis.[^17^, ^18^] This phase is complex, comprised of various chemical species with wide ranges of molecular weights and functional groups.[^19^, ^20^] Consequently, the precise identification, quantification, and characterization of LO, crucial for understanding their chemical behavior and functionalities, remain challenges for scientific research. Therefore, tackling these analytical challenges becomes essential for maximizing the potential of lignin‐based resources and looking for a way towards a more sustainable energy landscape.

An interesting solution to these challenges involves the development of synthetic lignin oligomers (SLO) in sufficient quantities that can serve as reference standards for identifying and characterizing LO. SLO with well‐defined structures and properties provide a means to establish robust correlations between molecular structure and analytical responses, enabling more accurate interpretation of experimental Data and more accurate identification/quantification. Moreover, the availability of SLO holds broader implications beyond structural identification, as they would facilitate in‐depth investigations into the phenomenology of lignin thermochemical conversion reactions. By studying SLO under these conversion processes, researchers can obtain valuable insights into reaction mechanisms, kinetics, product distribution, and the influence of various operating conditions.

The size of an SLO is a critical metric. While naturally isolated lignins were found to contain LO with size ranges of 7–25 monomers (BC of black liquor)^[21]^ or 40–65 monomers (solid residue),^[22]^ it is difficult to synthesize SLO with comparable sizes that are stable enough to be the subject of further study. A good example is the work by Katahira et al.,^[23]^ where SLO (≤4 monomers) were synthesized, and their hydrothermal depolymerization was investigated.

The objective of this work is to provide valuable information on the synthesis and characterization of SLO, which we consider paramount to enable the use of lignin as a crucial resource in the search for greener alternatives and foster advances in sustainable energy production. In addition, this article makes an in‐depth and critical analysis of the analytical techniques and various methodologies for synthesizing molecules that are used as models of lignin structures and could be viable as standards in the range of monomer units from 2 to 8. Strategies are proposed to address the existing knowledge gap through the development and use of SLO, focusing on the most feasible methods described in the literature for their synthesis. By exploring and adopting these approaches, researchers can advance the understanding of LO and overcome the challenges associated with its identification, quantification, and characterization of lignin and its derived products.

Section 1 will focus on the chemical nature of natural (section 1.1) and technical lignin (section 1.2) as well as the various depolymerization methods to obtain LO (section 1.3) and fractionation of LO‐rich liquid products (section 1.4). Section 2 summarizes characterization techniques applied to LO and SLO, while Section 3 discusses the synthesis of standardized SLO with different potential uses. To finish, Section 4 briefly discusses still existent gaps and proposes future research directions.

### 1.1 Nature of Lignin Oligomers

**Figure 2:**
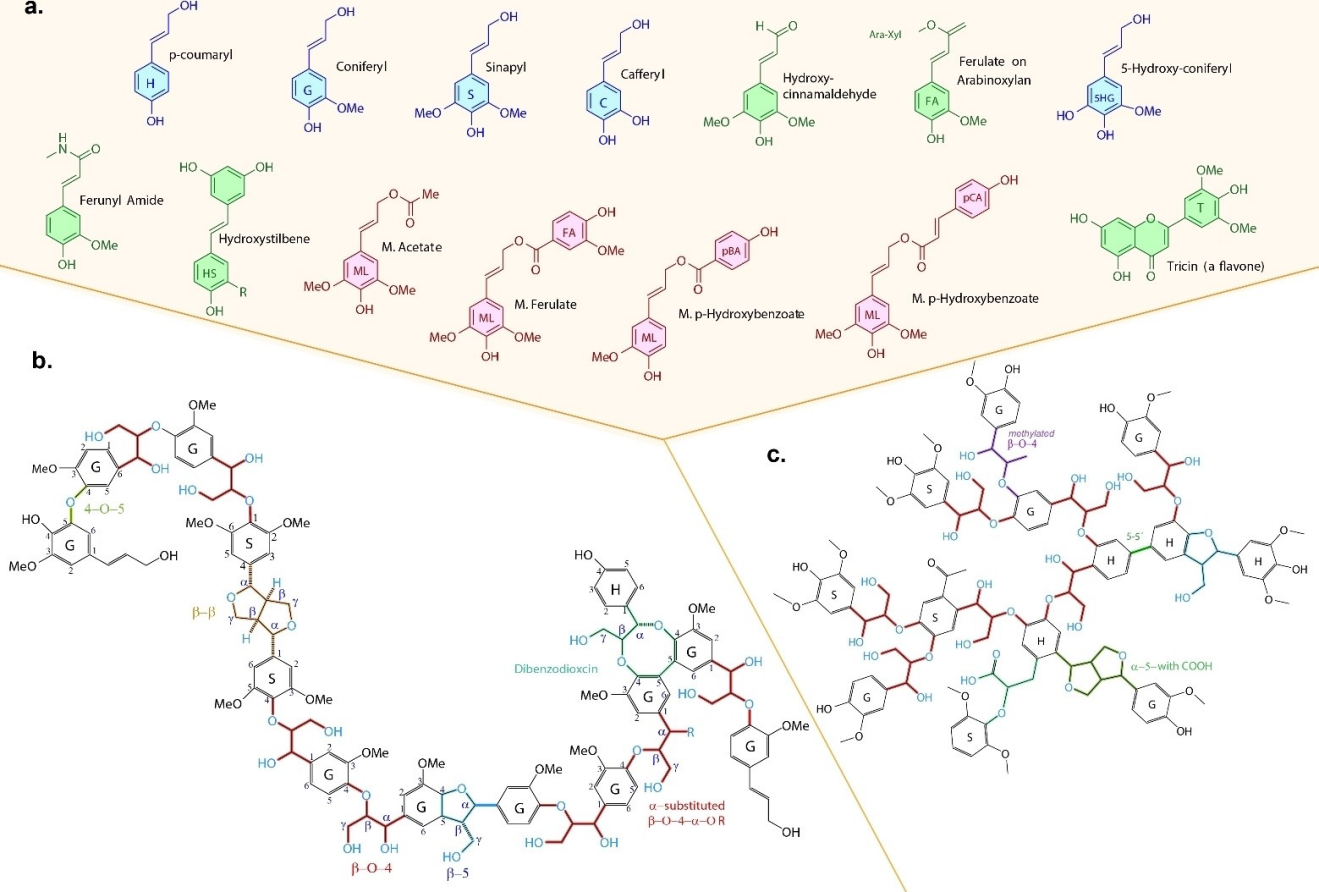
Structures of a) constituent monomer units. Adapted from Ref. [29], Copyright (2021), with permission from Elsevier. b) lignin structure. Adapted from Ref. [25] Copyright (2020), with permission from Wiley, and c) linkages of lignin oligomers. Adapted from Ref. [37] Copyright (2023), with permission from KIT.

Lignin is a highly complex amorphous polyaromatic polymer generated by nature in plants, which consists of phenylpropanoid subunits linked by a wide variety of C‐O‐C (β‐O‐4, α‐O‐4, 4‐O‐5) and C‐C (β‐1, β‐ β, 5–5) bonds.^[24]^ These compounds primarily originate from the combinatory radical coupling of the monolignols (Figure 2): coniferyl alcohol known as guaiacyl (G) with around 95 % abundance in softwood, 25–50 in hardwood, and 35–80 in grasses, p‐coumaryl alcohol (H) abundant in grasses (5–35 %), hardwood (0–8 %) and less in softwood (0‐3 %), and sinapyl alcohol (S) absent in softwood, with 20–55 % and 45–75 % in grasses and hardwood respectively.[^25^, ^26^] According to literature, the β‐O‐4 linkage is the most prevalent connectivity observed in natural lignin, 50–60 % (wood) and ≤85 % (grasses), the β‐5 linkage is in second place with 3–12 % (wood) and 5–11 % (grasses), β‐1 1–9 %, β‐β 2–9 %, and 4‐O‐S 4‐9 % in wood (absent in grasses).[^25^, ^27^] Because β‐O‐4 linkage is more abundant, synthetic structures with this linkage are often used for depolymerization, modification, and catalysis studies.[^25^, ^28^] As reported by Ralph et al.^[29]^ Figure 2a shows the monolignols that constitute the structure of lignin and the LO, which are grouped into Hydroxy Cinnamyl alcohols (blue), conjugates (red), and other types (green). Figure 2b and c present theoretical structures for pyrolytic lignin. Lahiye et al.^[25]^ devised a linear molecule highlighting important linkages, while Fonseca et al.[37] devised the structure specific to a wheat straw based on complex analytics provided by different sources.[^30^, ^31^, ^32^]

To address the challenges in lignin and its derivatives characterization and enhance the understanding of their chemical structure, it is necessary to develop SLO in sufficient quantities that can serve as reference standards for identifying and characterizing LO. SLO with well‐defined structures and properties provide a means to establish robust correlations between molecular structure and analytical responses, enabling more accurate interpretation of experimental data and more accurate identification/quantification. Moreover, the availability of SLO holds broader implications beyond structural identification. SLO can facilitate in‐depth investigations into the phenomenology of lignin thermochemical conversion reactions. By studying SLO under these conversion processes, researchers can obtain valuable insights into reaction mechanisms, kinetics, product distribution, and the influence of various operating conditions.

Previously, SLO up to 4 monomers have been synthesized and their hydrothermal depolymerization was investigated;[^21^, ^23^] however, in BC from Black liquor LO of about 7–25 monomer units are found and in solid residue LO in a size between 40–65 monomer units are observed,^[22]^ which emphasizes the need appropriate SLO in sufficient molecular size.

### 1.2 Production of Technical Lignin

Lignin fractions can be isolated from the original lignocellulosic matrix using different chemical treatments. The washed isolated lignin from these treatments has acquired further commercial interest in the last few decades and is often called technical lignin.

**Figure 3:**
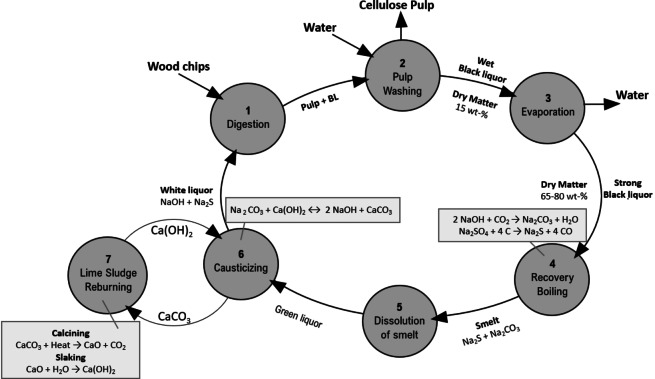
Main material flows in a Kraft pulp mill process. Adapted from Ref. [38] Copyright (1996), with permission from Elsevier.

Technical lignin is available in large quantities as residue from producing cellulose fibers, e. g., to produce paper. However, it is also an emerging by‐product from the pre‐treatment of lignocellulosic material to enable sugar‐based microbial processes, such as ethanol production from sugarcane bagasse or straw. The most used process for the production of cellulose worldwide is Kraft pulping, where wood chips are treated with cooking chemicals (sodium sulfide (Na_2_S) and sodium hydroxide (NaOH), that separate the cellulose pulp from the remaining lignocellulosic matrix components (hemicellulose and lignin). Salts collected in the cellulose pulp are removed by washing, while the black liquor, containing lignin and hemicellulose hydrolysis products, is concentrated by evaporation. The process is summarized in Figure 3.^[33]^

Traditionally, black liquor is concentrated by evaporation (step 3 in Figure 3), followed by its combustion to generate heat and power (step 4 in Figure 3. Said black liquor presents high inorganic, sulfur, and sodium contents. The composition of the organic phase depends on the type of wood used. Softwood black liquor contains G units, while hardwood products contain both S and G units, along with varying amounts of aliphatic carboxylic acids, methoxycatechols, and phenolics.^[33]^

Three commercial processes have been developed that replace step 4 of the Kraft process with complex methods to extract Kraft lignin: WestCaco (USA), LignoBoost® (Valmet, Sweden), and LignoForce® (FPInnovations/NORAM, Canada). They all involve the precipitation of dissolved lignin from black liquor and bleaching the lignin cake to produce technical lignin.^[34]^ However, technical Kraft lignin often has high sulfur content and low water solubility due to the Kraft treatment and bleaching process,[^35^, ^36^] which limits its use as a source for LO.

Another alternative to the Kraft process is sulfite pumping, targeting the production of bright, smooth cellulose pulp, making use of less corrosive agents like sulfurous acid (H_2_SO_3_) and its salts (like sodium sulfite, Na_2_SO_3_) to dissolve lignin. This treatment produces a wide range of lignosulfonates, which can be extracted from the filtrate and are water‐soluble throughout the entire pH range. Alternative valorization pathways are being studied for these materials.^[39]^

To overcome the negative characteristics of Kraft lignin, alternatives have been developed that do not rely on effluents from the Kraft process. One such alternative is soda‐alkali lignin, e. g., produced from sugarcane bagasse through alkaline hydrolysis to remove residual sugar content.^[35]^

*Organosolv* lignin is a newer alternative to Kraft lignin, which includes treating a lignocellulosic feedstock with an aqueous organic solvent at moderate temperatures. This method offers numerous advantages, namely using bio‐based solvents, low water pollution, and no odor. Moreover, the absence of sulfur and low acidity in the pulp and extract enable fermentation for syngas or high‐value bio‐product production.^[40]^ Commercial production of this material in a commercial scale is expected within the next decade, and a large increase in its market share is expected in the following years.^[41]^

A summary of the general delignification process can be found in Table 1.

**Table 1:** General delignification process description, including *M_N_* and *M_W_* for four types of technical lignin. Adapted from Ref.. [45] Copyright (2021) with permission from University of North Dakota.

Technical Lignin Production	Kraft lignin	Lignosulfonates	Soda‐alkali lignin	Organosolv lignin	Ref.
Delignification agents	NaOH, Na₂S	metal sulfite, SO_2_	NaOH	Organics: methanol, ethanol, acetic acid, formic acid (acid catalyst)	[46]
Process description	High pH (basic), 150–170 °C, ether bonds cleaved, some condensation, lignin precipitated at pH 5–7.5	Hydroxyl groups sulfonated, lignin is solubilized; 140–160 °C, pH 1.5–2.0; ether bonds cleaved, benzyl carbocations formed.	Primarily non‐wood biomass used; placed in pressurized reactor at 140–70 °C with about 15 wt. % alkali bases (usually NaOH)	Cleavage of ether bonds resulting in small MW species enables solubilization; 90–220 °C depending on the source, ethanol (if used) 25–75 % (v/v)	[39,47]
Chemical characterization	Sulfur content 1–2 wt. % as thiols; hydrophobic; C=C bonds, fewer ether bonds, fewer methoxy groups	Highly crosslinked product (carbocation‐π electrons attraction), 5 wt % sulfur as sulfonate groups (SO₃−), preserving solubility	High carboxylic acid content, very dispersed, must be heated to coagulate. No sulfur, very pure (no hemicellulose)	Smaller MW, higher purity, hydrophobic, no sulfur	[39]
M_N_ (g mol^−1^)^[a]^	Softwood 3000	Not available	Wheat straw 1700	Hardwood 800	[39]
M_W_ (g mol^−1^)^[b]^	1000–3000	20,000–50,000	800–3000	500–4000	[47]
Polydispersity Index^[c]^	2.5–3.5	6–8	2.5–3.5	1.3–4.0	[47]
Annual yearly production (kTon)	247.5–630^[d]^	1320–1800	82.5	33	[41,48–50]

[a]: Number molecular weight of a polymer: statistical average of the molecular weight of all chains in the polymer sample. [b]: Weight molecular weight of a polymer: molecular weight dispersion around the statistical average. [c]: Polydispersity index (PDI) is a measure of the broadness of the dispersion of molecular weights: PDI=M_W_/M_N_. [d]: The majority of the lignin concentrate produced during the Kraft process is incinerated in loco for the production of steam. This figure refers to the fraction of the concentrate that is available commercially.

### 1.3 Lignin Depolymerization Strategies for the Production of Lignin Oligomers

**Figure 4:**
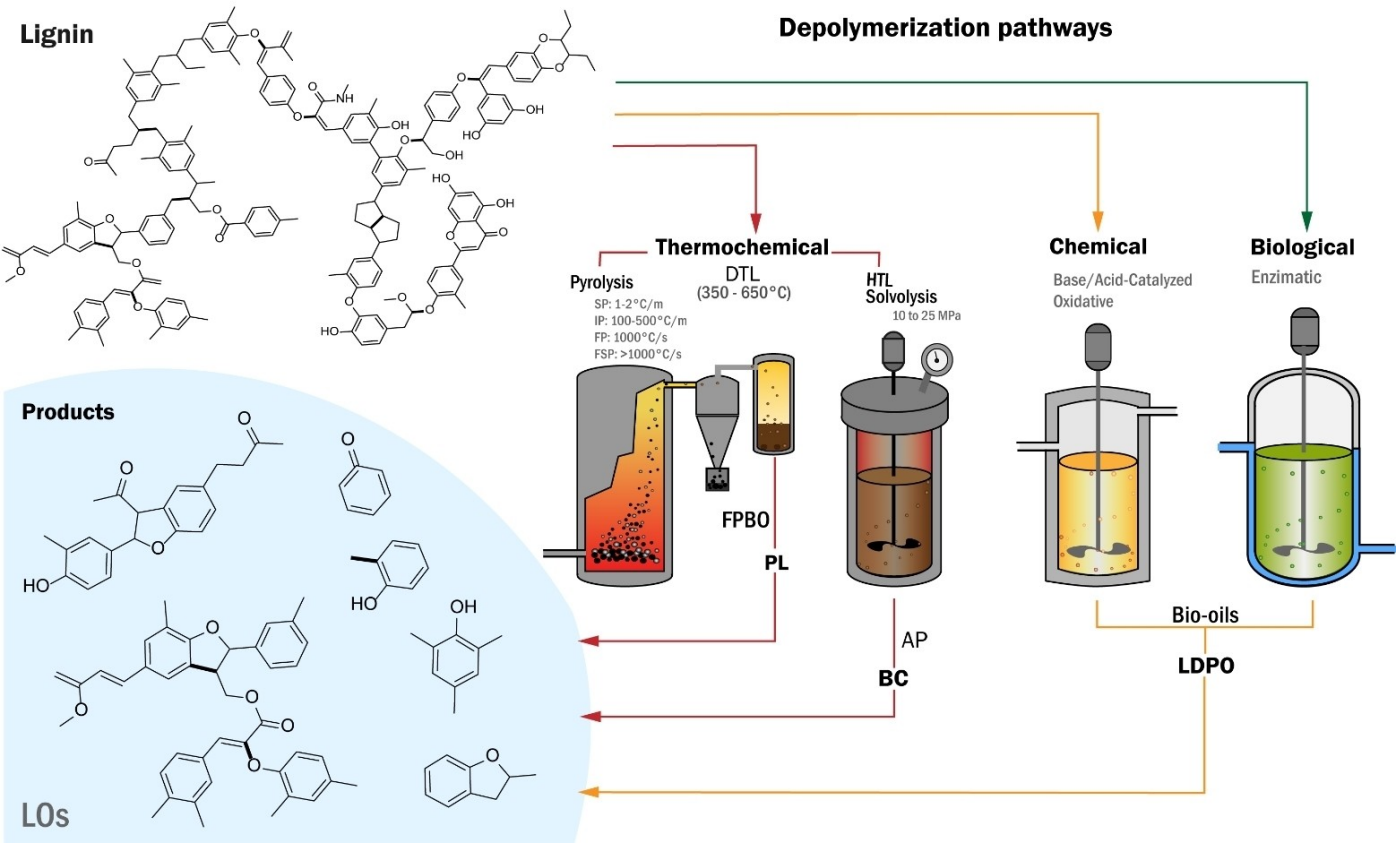
Depolymerization process to obtain liquid products rich in LO. (LDPO stands for lignin‐derived phenolics).

Lignocellulosic and lignin depolymerization involves breaking down complex polymers into smaller molecules.^[42]^ Strategies for depolymerization vary, with distinct characteristics and outcomes, focusing on producing LO and liquid products. Aryl ether bonds are easier to break than condensation bonds, making the products valuable for biofuels, biochemicals, and biomaterials. Lignin′s heterogeneous structure requires extreme conditions for depolymerization due to its resistance.[^13^, ^42^] Figure 4 shows three main routes of lignin depolymerization: thermochemical, chemical, and biological.

#### 1.3.1 Thermochemical Depolymerization

LO can be produced from the thermochemical depolymerization of lignocellulosic biomass or directly from lignin using heat and oxygen‐free reactions. Lignocellulosic biomass, primarily composed of hemicellulose, cellulose, and lignin, degrades differently during thermal decomposition, generating volatile compounds and biochar. Hemicellulose is the least thermally stable component, breaking down within the temperature range of 150–350 °C. Cellulose decomposes between 275–350 °C, while lignin decomposes over a wider range, from 180 to 500 °C.[^43^, ^44^] Several thermochemical processes permit using biomass and lignin to produce energy, chemicals, bio‐oils, and syngas.

These thermochemical processes involve physicochemical phenomena that occur at different scales whose understanding is necessary for scientists. For example, chemical conversion reactions occur on a microscale in which reactive molecules such as lignin are decomposed by thermal energy and the intervention of other present molecules or intermediates. This microscale provides information about the intrinsic chemical kinetics and the thermodynamic parameters that govern the processes.^[44]^ Hence, SLO must allow studies on this scale to help understand the real phenomenology of lignin depolymerization and LO generation.

***Direct thermochemical Liquefaction (DTL)*** involves solvent liquefaction and pyrolysis.^[51]^ In pyrolysis, the conversion typically occurs at temperatures between 350 °C and 650 °C; the liquid produced is known as bio‐oil (BO), solid (bio‐char), and gas (syngas.[^52^, ^53^] The depolymerization of lignin by pyrolysis typically starts with the cleavage of weak bonds (primary pyrolysis), such as ether bonds, including α‐O‐4 and β‐O‐4, at low temperatures (200 to 400 °C), followed by the decomposition of stronger bonds at temperatures above 400 °C (secondary pyrolysis), where the cleavage of the C‐C bonds is generated, and the LO and monomers are formed.[^12^, ^13^] The residence times vary depending on the specific subtype of pyrolysis, which is classified based on the heating rate employed.

***Slow pyrolysis (SP*** **)** involves heating rates of 1–2 °C/min with residence times ranging from several hours to approximately 24 hours, favoring char production with lesser amounts of BO and gas. ***Intermediate pyrolysis (IP)*** has heating rates of 100–500 °C/min and residence times from several minutes to an hour, balancing the yields of char, BO, and gas.^[54]^ ***Fast pyrolysis (FP)*** features heating rates of around 1000 °C/sec and residence times of less than 2 sec, favoring FPBO yield.^[55]^ ***Flash pyrolysis (FSP)*** uses very high heating rates exceeding 1000 °C/sec and ultra‐short residence times of less than 1 sec, achieving high amounts of FPBO with low water content and conversion efficiencies up to 75 %.^[51]^

***Solvent liquefaction***, also known as solvolysis, uses solvents at elevated temperatures (200 °C to 400 °C) and pressures (from 4 to 22 MPa).^[51]^ This process mimics the natural geological formation of fossil fuels but accelerates its occurrence within hours or even minutes. The main product is named BC, which typically has lower oxygen and water content, is more thermally stable, has higher viscosity, is less dense, and has a higher calorific value than BO.[^51^, ^56^] Common solvents include water (subcritical or supercritical), alcohols (e. g., methanol, ethanol), and organic solvents (e. g., acetone, ethylene glycol).^[56]^ The choice of solvent influences the efficiency and selectivity of the process. When supercritical solvents are used, it is known as Supercritical Liquefaction.^[57]^

***Hydrothermal Liquefaction (HTL)***, or hydrous pyrolysis, applies moderate temperatures (200 to 400 °C) and elevated pressures (10 to 25 MPa) in the presence of water or H_2_, converts the feedstock into BC (organic phase), aqueous extract (Aqueous phase), hydrochar (solid). Using other co‐solvents, such as ethanol and methanol, is also possible. Using catalysts, such as metal‐supported mesoporous materials (e. g., Ni‐Al/MCM‐41), can significantly enhance the depolymerization efficiency and improve the quality of BC. Catalysts aid in breaking down the complex lignin structure (β‐O‐4, C‐C, and inter‐unit linkages^[58]^ and deoxygenation processes, resulting in higher‐quality BC with lower oxygen content.^[59]^ The Aqueous phase contains water‐soluble organic compounds such as carboxylic acids, alcohols, and small phenolic compounds.^[60]^

#### 1.3.2 Chemical Catalytic Depolymerization

With these technologies, reactions can be better controlled, and therefore, the selectivity of the structures or bonds to be built can be improved.^[61]^ In addition, chemical catalytic depolymerization can be combined with temperatures to improve efficiency.^[62]^

**Base/Acid‐Catalyzed Depolymerization** involves the use of strong bases or acids to cleave the complex polymeric structure of lignin, resulting in the formation of phenolic monomers and oligomers.

***Acid‐catalyzed depolymerization*** is achieved using strong mineral acids (H_2_SO_4_ and HCl), organic acids (CH_3_COOH and HCOOH), and Lewis acids (AlCl_3_ and FeCl_3_).^[63]^ These acids interact with the oxygen atoms present in the lignin structure, facilitating the breaking of lignin bonds. This process is particularly effective at breaking α‐O‐4 and β‐O‐4 linkages. e. g., Zhuang Li et al.^[64]^ obtained guaiacol by directly breaking the Car‐Cα and Cβ‐O bonds. The cleavage mechanism is inferred by combining DFT calculations. An alternative approach is the ***Base‐catalyzed depolymerization (BCD)*** with strong bases (NaOH, KOH). Bases deprotonate hydroxyl groups in lignin, facilitating the cleavage of aryl‐ether bonds, particularly the β‐O‐4 linkages, and solubilizes lignin by forming alkali lignin salts. The resulting depolymerized lignin can be recovered by acidification or precipitation methods. For example, a high yield of phenols under mild conditions was obtained by combining catalyst (NaOH+NaAlO_2_) with Ni/ZrO_2._ ^[65]^

***Oxidative depolymerization*** involves the use of oxidizing agents such as hydrogen peroxide (H₂O₂), oxygen (O₂), ozone (O₃), nitric acid (HNO₃), and peracids to break down lignin. Metal catalysts, such as those based on vanadium, copper, and iron, can also be employed to enhance the reaction. Oxidizing agents attack the electron‐rich aromatic rings and ether linkages in lignin, including the cleavage of β‐O‐4 and other ether linkages, and secondary reactions generate further oxidation of primary products to carboxylic acids, aldehydes, ketones, and quinones.[^66^, ^67^] In this way, Figueirêdo et al.^[68]^ obtained oxygenated fragments and low molecular weight products suitable for high‐value chemical synthesis using O_3_ and Zaid Ahmad et al.^[69]^ achieved a yield of over 90 % in forming depolymerized Kraft lignin products through simultaneous oxidation and nitration.

#### 1.3.3 Biological Depolymerization

This method uses microorganisms and enzymes to break down lignin, mimicking natural processes. White‐rot fungi, like *P. chrysosporium*, are highly effective, producing enzymes such as lignin peroxidases (LiPs), manganese peroxidases (MnPs), and laccases.^[70]^ ***Enzymatic depolymerization*** utilizes specific enzymes, such as ligninolytic enzymes produced by fungi, that possess oxidative capabilities and can cleave lignin‘s interunit linkages. This approach takes advantage of the catalytic properties of enzymes to selectively cleave lignin bonds, offering a potentially more controlled and sustainable method. Kalia et al.^[71]^ discuss issues with enzyme dimension and low redox potential for laccases, which enable only surface oxidation of phenolic groups, allowing for surface modification but not complete consumption within a reasonable time frame. Caihong et al.,^[72]^ used microbial‐assisted enzymatic conversion of lignin into value‐added bioproducts. However, biological processes are generally slower and less efficient than chemical methods.

### 1.4 Fractionation of FPBO/BC

LO are contained in FPBO and BC, which are complex mixtures of a large variety of molecules. These complex mixtures can be separated into multiple fractions with reduced polydispersity through fractionation. Fractionation methods for the recovery of LO enriched fractions can be categorized into three main groups: (i) gradient acid precipitation, (ii) stepwise solvent fractionation, and (iii) membrane separation.^[73]^ This process serves two primary purposes: first, for analytical examination of its chemical structure, and second, to yield fractions with enhanced properties, facilitating their utilization. Among these purposes, solvent extraction is the most employed method due to its versatility and effectiveness.[^15^, ^74^]

FPBO and BC are recovered as dark brown, free‐flowing complex organic liquids with a wide number of chemical compounds^[75]^ and higher oxygen and water content (5‐30 wt. %) than fossil crude oil.[^76^, ^77^] The calorific potential of FPBO is similar to that of the feedstock (16–19 MJ h^−1^),[^75^, ^78^] while BC typically is almost twice as high (35‐30 MJ h^−1^).^[79]^ The high acidity (pH 2–3) of FPBO generates corrosivity on metals, and the presence of other oxygenated compounds generates “aging” making it an unstable product over time.[^78^, ^80^] In contrast, BC can be regarded as an equilibrium product with higher thermal stability.^[51]^ The complexity of the chemical composition of these products depends mainly on the biological feedstock and the thermochemical processing conditions. Due to these characteristics, FPBO currently mostly sees commercial use as boiler fuel,^[81]^ with emerging uses as gasification fuel,^[82]^ heavy‐duty fuel, refinery crude blend,^[83]^ automotive fuel,^[84]^ and source of chemicals. All these possible products are available in different degrees of industrial maturity.

Fractional condensation allows the recovery of multiple liquid phases of FPBO, increases the range of products recovered, and, at the same time, reduces the diversity of chemical compounds present in each final FPBO fraction.^[85]^ On the other hand, the production of BC does not enjoy the same level of technological maturity as that of FPBO, due to the higher difficulty in separating the products after reaction.^[86]^

**Figure 5:**
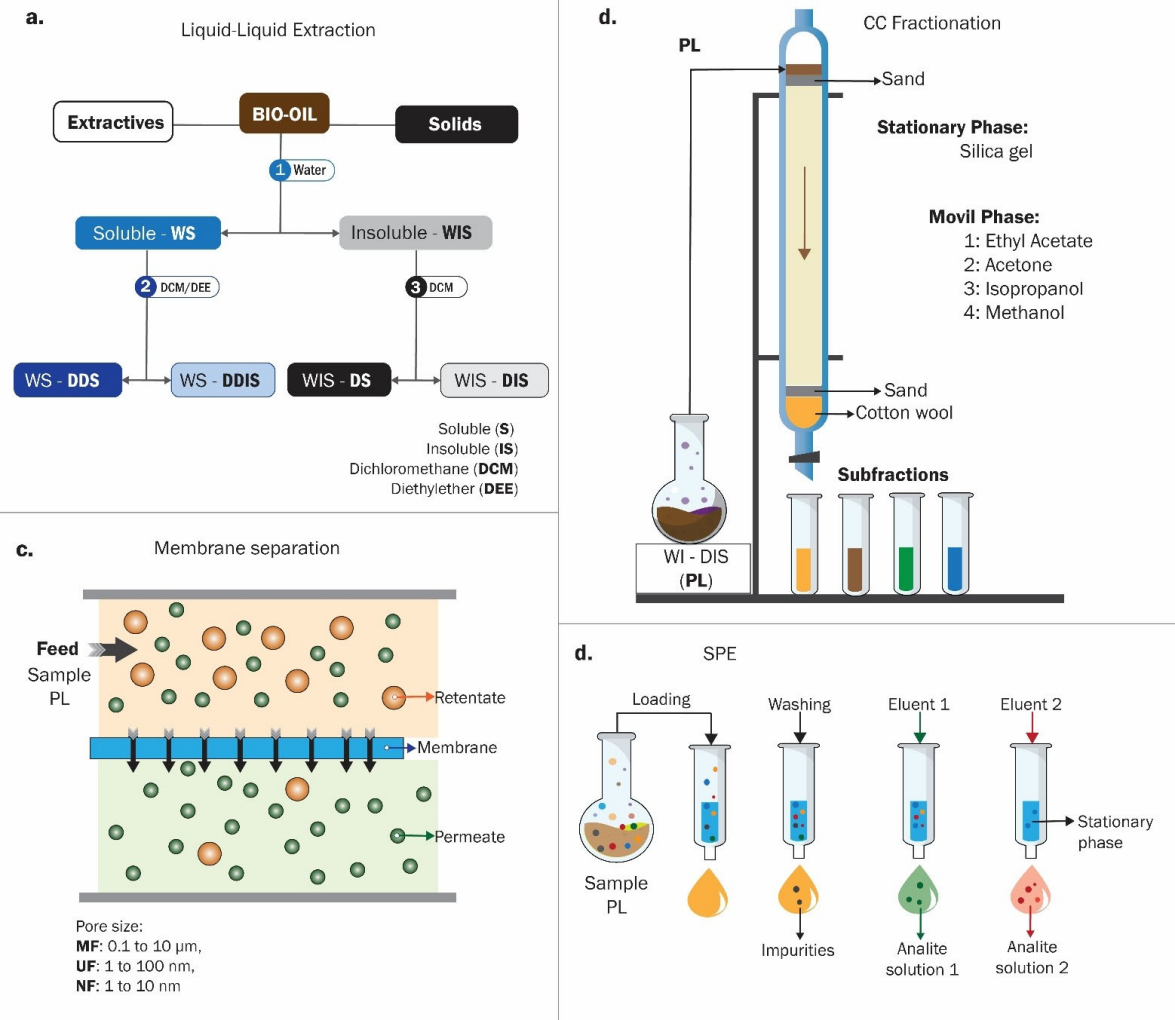
Chemical simplification of the FPBO composition (S: soluble, I: insoluble, DCM: dichloromethane, DEE: diethyl ether) a) Solvent fractionation by liquid‐liquid extraction., Adapted from Ref. [89] Copyright (2024), with permission from ACS. b) Continuation of column chromatography fractionation., c) Membrane separation., and d) Solid phase extraction (SPE) system.

For a reliable qualitative and quantitative analysis of complex samples, such as FPBO/BC, it is necessary to simplify the chemical composition of the samples using various separation techniques. In this way, a frequently used technique is fractionation by extraction with solvents of different polarities to facilitate the characterization of these liquids. Up to four different phases can be isolated for further analysis or processing. In Figure 5a, the oil is first separated into two phases: soluble and insoluble in water. These phases can be re‐fractionated with dichloromethane (DCM) or diethyl ether (DEE).^[87]^ The water‐insoluble phase constitutes from 3 to 29 % by weight of the FPBO,^[88]^ and each subsequent fraction can be associated with low molecular weight LO (DCM‐insoluble) and high molecular weight LO (DCM‐soluble).^[75]^ Rojas et al.^[89]^ recently addressed some of the challenges associated with the structural identity of this LO phase by modeling liquid‐liquid extraction using several surrogate structures from the literature.

Further research will indubitably increase the efficiency of the extraction process due to the considerable chemical diversity of the LO in terms of their chemical affinity with the solvents, leading to decreased selectivity. Other proper techniques include fractional distillation, centrifugation, and liquid‐liquid extraction continued with chromatographic techniques,[^90^, ^91^, ^92^] such as column chromatography (CC), as Figure 5b, and/or thin‐layer chromatography (preparative or semi‐preparative). Membrane separation techniques (Figure 5c), including microfiltration (MF), ultrafiltration (UF), and nanofiltration (NF), are also helpful methods for the fractionation of LO.[^93^, ^94^] These membranes are highly selectively separating structures with different molecular weights and functionalities. However, fouling is a significant challenge, as mixture solutions can lead to pore blockage and reduced membrane efficiency over time.^[93]^ In addition, solid‐phase extraction (SPE) with cartridges, as shown in Figure 5d, could be an up‐and‐coming technique for fractionating FPBO and facilitating the identification of LO. This technique has not yet been explored in the current literature to separate LO but is widely used in other very complex matrices, such as isolating natural products (secondary metabolites of various biomasses). The potential of this technique lies in the availability of commercially available stationary phases with distinctive chemical affinities, and it also requires a minimal volume of solvents and samples. Therefore, it would be a highly recommendable strategy for analytical purposes since this technique could allow for the efficient isolation of LO. Analytical techniques, both spectrophotometric and chromatographic, are helpful to characterize the LO of FPBO, and fractions are described in greater detail in Section 2.

## 2 Characterization of Lignin Oligomers

The complexity and diversity of oligomeric molecules found in FPBO and BC represent a great challenge for science in developing efficient analytical methods for identification and quantification. Spectroscopic and chromatographic techniques play a vital role in the structural elucidation of LO, as they allow the separation of compounds or groups of compounds, which facilitates the identification and characterization of molecules based on their chemical properties and molecular structures. Thermophysical techniques are also broadly applied for fundamental insights and break‐up of structures. Although notable progress has been made, as detailed below, there is still a considerable distance to be covered.

### 2.1 Spectroscopic Techniques

#### 2.1.1 Nuclear Magnetic Resonance (NMR)

NMR spectroscopy is an advanced characterization tool that enables the identification of the atomic‐level molecular structure of a substance, it provides valuable structural information, including the connectivity of atoms and the arrangement of functional groups.[^95^, ^96^] NMR 1D (^1^H‐NMR, and ^13^C‐NMR), provides information about the chemical shifts and intensities of signals in a molecule, and NMR 2D (Correlation Spectroscopy (COSY), Heteronuclear Single Quantum Coherence (HSQC), and Heteronuclear Multiple Bond Correlation (HMBC)), involves the correlation of two NMR frequencies, providing additional information about the connectivity and spatial arrangement of atoms in a molecule. Both techniques have been widely used in the analysis of LO and SLO; however, NMR 2D provides more accurate information to identify the kind of linkages, substitution patterns, aromatic ring analysis, quantification of structural features, and molecular weight analysis.[^95^, ^97^, ^98^, ^99^] NMR is useful for semi‐quantifying the individual G/S/H units and the G/S ratio, but the absolute quantization of the links and Dangling units remains challenging as signal integrations can be influenced by many factors such as structural heterogeneity, conformational flexibility, signal overlap, and sensitivity.[^95^, ^100^, ^101^] Table 2 summarizes recent results found in the literature, which are discussed below.

**Table 2:** Spectroscopic Techniques are useful to analyse the LO and SLO.

Entry	Technique	Sample or analytes	Characteristics	Findings	Ref.
1	^31^P NMR and ^13^C solid state NMR	HFPBO from Black liquor	Detection of functional groups in LO (COOH, H and G type, 5–5’, beta 5, C 5, aliphatic OH)	In solids and liquid products: raising of alkylated and methylated structures and decrease of arylethers and carboxy groups with temperature in HTL, Monomers and Oligomers pass comparable alkylation reactions	[141]
2	HSQC_0_ NMR (gradient‐selective method)	Isolated lignin with Ether contents of 6–46 % and SLO	G and S homopolymers GS: 70 mol % syringyl and 30 mol % guaiacyl units β‐O‐4 linkage (Cα, Cβ, and Cγ)	Ether bonds no longer appear to be randomly distributed in isolated lignin as in native lignin. It was an effective method for the quantification of chemical functionalities using SLO models able to analyze LO	[104]
3	2D HSQC NMR	Isolated lignins and SLO (hardwood and softwood)	Quantitative identification: β‐O‐4, β–β and β‐5 linkages Units: G, S, and S/G *Hardwood SLO*: S/G units with β‐O‐4 y β–β linkages *Softwood SLO*: β‐O‐4, β–β, β‐5 5–5 units and phenolic groups	SLO containing all three or four units were identified: – Two octamers (Softwood) – One tetramer (Hardwood)	[105]
4	MALDI‐FT‐ICR MS HSQC NMR	Wood lignin and SLO are separated by CC and thin‐layer chromatography (TLC)	HSQC NMR confirmed the CC separation of SLO types β‐β′, β‐5′, and β‐O‐4′. Dominance of the sodium adduct cation for the dimers (β‐O‐4′): m/z 399.35, (β‐5′): m/z381.37, and (β‐β′): m/z 381.37. Trimer (β‐O‐4′) m/z 595 and higher m/z 1000 y 1500. Mass increments of 196 (formation of β‐O‐4′), 178 (β‐5′ and β‐β′), and 180 (nucleophilic addition of coniferyl alcohol to a quinone methide intermediate)	Higher masses were detected that still cannot be deciphered. Both techniques provided fundamental information about lignin polymerization and structural populations based on SLO molecules.	[127]
5	APPI FT‐ICR MS, ^13^C, 1H‐^13^C HSQC, ^31^P NMR	Pristine lignin, Kraft, and oxidized products (modified LO)	Main signals: until m/z 500, range *m/z* 400–500, below: m/z 350. Ox classes: O_4_ and O_5_ (diphenolic structures), O_2_ and O_3_ (monomers) Until O_12_ (trimers and tetramers) in natural lignin samples Mainly: G units, S/G: 0.24 Semi‐quantitative contents: β‐O‐4, β‐β, and β‐5 linkages Aldehyde signals: δH/δC 9.8ppm/191.2ppm	Consistent results between the techniques used Mild oxidation conditions allowed to preserve of original lignin structures and reactivity partially depolymerized Medium‐sized molecules suitable for the preparation of phenolic resins.	[106,107]
6	1D ^1^H‐^13^C‐NMR 2D ^1^H‐^13^C‐HSQC 2D ^1^H‐^13^C‐HMBC 2D ^1^H‐^13^C‐LR‐HSQMBC 2D ^1^H‐^1^H ROESY MALDI‐TOF‐MS	SLO acetylated and non‐acetylated forms using good and aqueous poor (D_2_O) solvents (D_2_O, and DMSO‐d_6_: 0–90 % volume of acetic acid‐*d_4_* buffer in D_2_O (pD 5.0))	Trimer and tetramer models (SLO 1) The linkages formed were only of the β‐O‐4 type ROE Signals: between Terminal C1‐B 6 and A β‐C 5(I) in DMSO‐d6 (Short‐chain SLO)	SLO is composed of only erythroisomers. SLO showed similarly folded conformations in both solvents, D_2_O and DMSO‐d_6_ solvents	[109]
7	FT IR FT‐ICR‐MS [ESI (−) FT‐ICR)]	Kraft lignin with enzymatic treatment	FTIR: 14 bond signals assigned to groups (656 to 2938 cm^−1^) Groups assigned: – Lignin: O/C:0.3‐0.7 and H/C: 1–1.5 – Condensed aromatics: O/C < 0.25 and H/C: 0.5–1.0. – Fatty acids or esters/lipids: O/C < 0.2 and H/C: 1.6–2.0 – Carbohydrates: O/C: 0.8–1.0 and H/C: 1.65–2.0	Over 3350 peaks (S/N > 4), with an MS resolution of ˜300,000 at m/z 400, highlight the sample‘s high chemical complexity. In total, 3137 elemental formulas were assigned.	[121]
8	GALDI‐FT‐ICR‐MS [ESI‐, liquid GALDI‐, and solid GALDI‐FT‐ICR‐MS]	Alkali lignin (Sigma‐Aldrich) Indulin AT, and Lignoboost lignin	Indulin AT: 458,12 Da Lignoboost: 448,76 Da Optimized ESI‐MS: to ionize lignins in a m/z range between 200 and 850 Da. GALDI‐MS: up to 650 Da. Optimized solid GALDI‐MS: highest m/z range from 200 to 1000 Da	Three FT‐ICR‐MS methods for lignin were developed and optimized able to analyze from monomers to pentamers. To obtain more detailed structural information, tandem MS techniques are recommended.	[130]
9	2D HSQC NMR Py‐GC/MS MALDI–FT‐ICR‐MS	Lignin extracted from biomass	S/G ratio estimated: 0.89 (Py‐GC/MS) and 0.86 (NMR) O/C ratio: 0.2–0.5 H /C ratio: 0.8–1.3 Dimers to hexamers β‐O‐4, β‐β, and β‐1, with H, G, and S units.	100 lignin structures purposed by stochastic lignin simulation based on the experimental results Stochastic modeling of complex macromolecules directed by MALDI‐FT‐ICR MS could be adapted to other macromolecules such as SLO	[123]
10	SEC 2D ^1^H‐^1^H TOCSY, 2D ^1^H‐^1^H ROESY MALDI‐TOF‐MS	SLO β‐ O ‐4 type obtained by methods 24,103,138	SLO short‐chain trimmer (Mn: 2.27) SLO long‐chain tetramer (Mn: 4.49) The SLOs resulted in low polydispersity with only β‐ O ‐4 bonds and erythro‐isomers.	Homogeneous SLO were synthesized to allow for the complete assignment of the NMR signals. Using MALDI peaks of sodium adducts of trimer and tetramer identified	[109]
11	Liquid‐state ^1^H NMR and ^13^C NMR FT‐ICR MS GALDI‐FT‐ICR‐MS GALDI‐MS/MS ESI‐MS/MS	Fractionated LignoBoost lignins (LBL)	Structural suggestions for precursor ions: 493.15 Da, C_27_H_25_O_9_ 507.17 Da, (C_28_H_27_O_9_, 567.19 Da, (C_30_H_31_O_11_, 627.22 Da, C_36_H_35_O_10,_ 508 Da, C_28_H_28_O_9,_ 510 Da, C_27_H_26_O_10_ 510 Da, C_28_H_30_O_9_	MS/MS results are in good alignment with the NMR. Precursor ions predominantly consist of G units, which are connected via β‐5′ bonds (absence of [M‐H‐H_2_O‐CH_2_O] – fragment ion). Small quantities of β‐O‐4′ and β–β′. Dominant terminal groups: methoxy, carboxy, and primary hydroxy groups	[133]
12	HPLC‐ HRAM‐MS with ESI Q‐Exactive Orbitrap MS	SLO sintetized by methods[^136^, ^137^]	SLOs β‐O‐4 and a, b‐diaryl ether linkage. SLO incorporates functional groups phenolic alcohol, aryl glycerol β‐aryl ether bond, and an unsaturated side chain.	SLO incorporates functional groups phenolic alcohol, aryl glycerol β‐aryl ether bond, and an unsaturated side chain. β‐O‐4 shows higher ion response and less in‐source fragmentation in negative ion mode than α,β‐diaryl ether.	[137,138]
13	H‐ESI‐FT‐Orbitrap MS [negative and positive ion UHRMS analyses]	FPBOs	Classes identified: O_2_, O_3_, O_4_, O_5_, N_1_O_2_, N_1_O_3_, N_1_O_4_, N_2_O_2_, N_2_O_3_, N_2_O_4_, N_3_O_2_, N_3_O_3_,	Limited scanning range: 100–700 Da	[133]
14	APPI‐Orbitrap HRMS	Lignins SDL and HDL isolated from wood.	LOs with varying degrees of polymerization, including β‐O‐4, β‐β, and β‐5 linkages bonds with G, S, and H units.	Negative ion mode APPI predominantly leads to the cleavage of ether β‐O‐4 bonds, causing significant fragmentation. Lignin‐carbohydrate complexes were located in the van Krevelen diagram region with H/C ratios of 1.2–1.8 and O/C ratios of 0.4–1.0.	[139]

*ROESY: Rotating‐frame Overhauser Effect Spectroscopy, TOCSY: Total Correlation Spectroscopy, APPI‐Orbitrap HRMS: Atmospheric Pressure Photoionization (APPI) coupled Orbitrap High‐Resolution Mass Spectrometry (HRMS), UHRMS: Ultra‐High‐Resolution, Mass Spectrometry.

Another valuable method for analyzing functional oxygen‐containing groups in LO is ^31^P‐NMR.^[102]^ spectroscopy. This technique quantifies the hydroxyl (OH) groups in lignin after its derivatization, allowing for a more detailed characterization of its structure (Entry 1). The dry lignin sample is dissolved and combined with pyridine, N,N‐dimethylformamide, deuterated chloroform (CDCl_3_), an internal standard (IS), and a relaxation reagent. It is then phosphorylated in a pyridine and CDCl_3_ mixture. The NMR spectrum of the sample and IS derivatives are obtained by liquid NMR spectroscopy and the OH groups by comparing the integrated signals of lignin and the IS.

On the other hand, in Entry 2, using a modified HSQC‐NMR method (HSQC_0_), the functionalities of homopolymers (G) and copolymers (GS) synthesized by the same previous Kishimoto method^[103]^ were quantified. Based on these results, the depolymerization yield of natural lignin was subsequently predicted using a 2‐D HSQC NMR spectra based model.^[104]^

Lancefield and Westwood (Entry 3),^[105]^ used HSQC NMR and Gel Permeation Chromatography (GPC) to identify that the SLO comprised (SG) units and β‐O‐4 and β‐β linkages, representing lignin from a hardwood model with phenolic groups. More links between units have only limited C‐H or C‐O correlation signals, independent of individual molecular structures. Therefore, the total relative distribution of a specific link between units or a final unit can be quantified/semi‐quantified for the entire sample as Entry 5.[^106^, ^107^] A disadvantage of this spectroscopic technique is that only general information about these molecular structures can be obtained. Consequently, it is challenging to investigate differences in the distribution of specific structures in specific classes,^[96]^ in addition to NMR sensitivity limits, which are overcome by two‐dimensional gas Chromatography‐Mass Spectrometry (GC x GC‐MS)^[106]^ This technique will be emphasized in the subsection 2.1.2.

In NMR, having LO standards or model compounds for analyzing natural LO in thermochemical processes offers several advantages.^[108]^ They allow for the structural characterization of LO,^[109]^ aiding in identifying specific features and understanding the impact of thermochemical processes or solvent effects (Entry 6). Additionally, they facilitate the quantification and monitoring of LO during these processes, enabling a quantitative assessment of lignin transformation. Standards or model compounds also assist in process optimization by providing insights into reaction kinetics and optimal operating conditions. In addition, they support the development and validation of specific analytical methods to analyze LO in liquid depolymerization products, guaranteeing more precise and reliable measurements using NMR and combinations with other analytical techniques.

#### 2.1.2 Fourier‐Transform Infrared (FTIR)

FTIR obtains the infrared spectrum (IR) of absorption, emission, or photoconductivity in samples that may be solid, liquid, or gas. This technique can identify functional groups such as hydroxyl groups (O‐H), methoxy groups (C‐O), carbonyl groups (C=O), and aromatic rings (C=C). It also provides information about the types and distribution of linkages in lignin, including guaiacyl (G), syringyl (S), and p‐hydroxyphenyl (H) units.^[110]^ FTIR spectra can be simulated using Density Functional Theory (DFT), which helps predict the spectra of theoretical structures.^[111]^ For example, one can predict the spectrum for a specific SLO and validate it with the created structure. After validation, it is possible to predict more spectra of this structure, considering variations in functional groups. However, there are some limitations to consider. Accurate analysis requires relatively pure samples because impurities can affect the spectrum,^[112]^ which is challenging with FPBO fractions. While FTIR is excellent for qualitative analysis, quantitative analysis can be complex due to varying baseline corrections and peak deconvolution. Finally, the accuracy of FTIR analysis depends on proper calibration with standards, which may not be available for all lignin derivatives and pyrolytic products.

#### 2.1.3 Ultraviolet‐Visible (UV‐Vis)

UV‐Vis spectroscopy is an effective method for determining the concentration of technical lignins in a solution with solvents by focusing on specific wavelengths such as 280 or 440 nm.^[113]^ Additionally, Ohra‐aho et al.^[114]^ uses this technique at 412 nm to determine the content of formaldehyde and formaldehyde derivatives (methylene glycol and oligomers) in FPBO and its fractions. Considering that LO have a high degree of aromaticity and conjugation, UV‐Vis is particularly suitable for their analysis. This suitability is why, in High‐Performance Liquid Chromatography (HPLC) or GPC, UV‐Vis detectors (single wavelength) and DaD/PDA detectors, which measure the absorbance of UV and visible light across a broad range of wavelengths simultaneously, are often used. However, the complex mixtures present in FPBO fractions result in spectra that are often broad and overlapping, complicating the analysis. For quantitative purposes, known standards are also required.

#### 2.1.4 Mass Spectrometry (MS)

MS techniques involve ionizing chemical structures to produce charged molecules or fragments and measuring their mass‐to‐charge ratios. The steps include ionization, mass analysis, and detection. MS is an efficient technique that has played a crucial role in analyzing and understanding complex natural organic matter (NOM)‐type molecules, such as LO.^[115]^ The fragmentation patterns observed in MS can help elucidate their structures, offering insights into their chemical composition and the types of linkages present.

This technology enables the detection of mono‐, di‐, and trimers following chromatographic separation. It is commonly used with gas chromatography and liquid chromatography coupled to MS (GC‐MS and LC‐MS) in one or two dimensions (MS and MS/MS).

Obtaining an MS spectrum of an analyte (chemical structure of interest in an analytical procedure) involves several key steps. The preparation of samples from complex mixtures involves extraction and purification processes to isolate the desired molecules from complex matrices,[^116^, ^117^] as well as derivatization, depending on the nature of the sample and the specific requirements of the MS technique used.^[118]^ The ionization of the analyte in the mass spectrometer is a critical step, as only ions can be manipulated and detected in a mass spectrometer. There are several ionization techniques:[^119^, ^120^]

*Gas phase methods* such as Electron Ionization (EI), Chemical Ionization (CI), Collision‐Induced Dissociation (CID), Direct Analysis in Real‐Time (DART), and Inductively Coupled Plasma (ICP).^[119]^ *Desorption methods* like Matrix‐Assisted Laser Desorption Ionization (MALDI), Fast Atom Bombardment (FAB), thermal ionization sources, plasma ionization sources, Liquid Metal Ion Sources (LMIS), and *Spray methods*, including Electrospray Ionization (ESI) and Desorption Electrospray Ionization (DESI).^[120]^

##### 2.1.4.1 FT‐ICR MS

*Fourier‐Transform Ion Cyclotron Resonance Mass Spectrometry* (FT‐ICR MS) is a robust analytical technique for analyzing complex chemical structures like the LO and SLO, as presented in Table 2. Based on their mass‐to‐charge ratio (*m/z*): monomeric compounds are often found in the range of 100–250 *m/z*, dimeric in the range of *250–400 m/z*, and larger oligomers from 400 *m/z* (Entry 7).^[121]^ With this technique, it is possible to obtain mass spectra that provide information about the compound′s molecular masses, elemental compositions, and structural characteristics. The high mass resolution of FT‐ICR MS helps to resolve closely spaced peaks, allowing differentiation of isomeric structures and identification of individual molecules.^[122]^ The results can be used to derive the molecular formula *C_x_H_y_O_z_* of the compounds.^[123]^ Subsequently, the weighted average of the number of carbon atoms, the number of hydrogen atoms, the number of oxygen atoms, and the number of sulfur atoms can be calculated to generate van Krevelen diagrams, Kendrick mass defect plots, and carbon number versus double bond equivalent (DBE) plots are used to improve the analysis and interpretation of the acquired Data.^[107]^ In other studies, the carbon number was plotted against DBE to identify the groups corresponding to dimers (20C, 10 DBE), trimers (30 C, 15 DBE), tetramers (39C, 21 DBE), and pentamers (49C, 25 DBE).[^124^, ^125^] Atmospheric Pressure Photoionization (APPI) was also utilized for the FT‐ICR‐MS characterization, specifically targeting species with molecular weights ranging between 150 and 700 Da.^[126]^ It is essential to consider that FT‐ICR MS is crucial for distinguishing between isomers.^[123]^ Therefore, it is recommended that this technique be combined with others, computational theoretical analysis, and MS techniques to achieve more robust results.

FT‐ICR MS can be combined with various ionization techniques, such as ESI, APPI, and MALDI,[^107^, ^127^] enhancing its versatility for analyzing fractions of LO and SLO. The results obtained from FT‐ICR MS are precious for molecular simulation and modeling to predict the chemical structures. However, FT‐ICR MS instruments are expensive and complex, requiring significant investment and expertise. The necessity for an ultra‐high vacuum environment can also pose a technical limitation, complicating maintenance and operation.^[128]^

Another challenge is the impact of impurities in the samples, a common problem in FPBO and BC fractions, which can cause spectral interferences. This is particularly problematic in complex mixtures where numerous compounds can obscure the signals of the target analytes. Impurities can contribute to the formation of unexpected adducts or fragments, complicating the determination of the molecular formula. Ion suppression is when the ionization efficiency of the target compounds is reduced.[^128^, ^129^] Additionally, impurities increase the complexity of data processing and analysis, presenting significant challenges.

##### 2.1.4.2 GALDI‐MS and MALDI‐FT‐ICR MS

Solid/Liquid Gallium‐Desorption/Ionization Mass Spectrometry (GALDI‐MS) and Matrix‐Assisted Laser Desorption/Ionization Fourier‐Transform Ion Cyclotron Resonance Mass Spectrometry (MALDI‐FT‐ICR MS) have also demonstrated reasonable progress in the characterization of LO in combination with other techniques.

FT‐ICR‐MS combined with ESI‐ and solid/liquid GALDI‐MS techniques (Entry 8) determined that Kraft and Lignoboost lignins are predominantly composed of monomers up to pentamers. Among the methods employed, solid GALDI‐MS emerges as the most suitable approach, not only for analyzing larger polar lignin oligomers but also examining less polar compounds with higher DBE values.^[130]^ Terrell et al.^[123]^ present a novel strategy that combines a stochastic computational model of complex macromolecules directed by MALDI‐FT‐ICR MS (Entry 9). This approach can be adapted to SLO to generate a molecular library that supports assigning potential candidate structures to the compounds detected during FT‐ICR MS analysis. NMR 1D and 2D, combined with SEC and MALDI‐TOF‐MS, were employed to characterize the short‐chain (M_w_: 613 Da) and long‐chain (M_w_: 809 Da) SLO models obtained through the Katahira method^[23]^ (Entry 10, described in Section 3). The SLO showed similarly folded conformations in both solvents, but a slightly more compact conformation was observed in D_2_O (Deuterium Oxide) compared to DMSO‐d6 (Deuterated Dimethyl Sulfoxide).^[109]^ Similarly, three SLOs were obtained by Kishimoto et al.,^[131]^ and Lancefield et al.^[132]^ were characterized as β‐O‐4 type. It is important to consider that for a complete assignment of NMR signals NOESY (Nuclear Overhauser Effect Spectroscopy) and ROESY (Rotating‐frame Overhauser Effect Spectroscopy), homogeneous chemical structures of low polydispersity are required, as is the case of the SLO models used in this study. However, these methods show limitations for the analysis of LO because lignin comprises irregular internal linkages, providing insufficient information on the resulting spectra‐.^[109]^

In Entry 11, ESI‐FT‐ICR‐MS and GALDI‐FT‐ICR‐MS characterize soluble and less‐soluble LO. ESI is more suitable for soluble lignin molecules, while GALDI is better for larger, less‐polar LO. Tandem MS (MS/MS), provides detailed structural information, confirming the presence of specific functional groups and linkages such as methoxy, carboxy, and hydroxy groups. The study used collision‐induced dissociation (CID) to fragment ions and analyze their structure.^[133]^

##### 2.1.4.3 H‐ESI‐FT‐Orbitrap MS

The characterization of the liquid products is carried out using High‐Resolution Electrospray Ionization Fourier‐Transform Orbitrap Mass Spectrometry (H‐ESI‐FT‐Orbitrap MS), which allows the identification of compounds with high polarity and/or high molecular mass.^[134]^ Moreover, several chemical oxidation treatments offer valuable insights into the structural characterization of lignin samples. For instance, cupric oxide (CuO) selectively breaks ether linkages between different monomeric units while leaving the aromatic rings intact, enabling the determination of the structural moieties (H/G/S) present in the samples. Amit et al.^[35]^ utilized this technique to characterize isolated Kraft and Alkali lignin, detecting common products such as vanillin (>80 %), acetovanillone, guaiacol, and vinyl guaiacol, all belonging to the G‐moiety category. Notably, Kraft lignin showed the presence of homovanillic acid (G), while Alkali lignin contained p‐cresol (H). However, S moieties were detected in either case.

SLO obtained by the methods,[^135^, ^136^, ^137^] were sequenced and characterized using Q‐Exactive Orbitrap MS with an ESI source (Entry 12). Chloride adduct ionization tandem provides sequence‐specific fragment ions, enabling the identification and characterization of lignin dimers and trimers with unambiguous sequence information:^[138]^ H‐(β‐O‐4)‐H, H‐(β‐O‐4)‐G, H‐(β‐O‐4)‐S, G‐(β‐O‐4)‐H, G‐(β‐O‐4)‐G, G‐(β‐O‐4)‐S, S‐(β‐O‐4)‐H, S‐(β‐O‐4)‐G, S‐(β‐O‐4)‐S, and a trimer H‐(β‐O‐4)‐G‐(β‐O‐4)‐S, G‐(4‐O‐α)‐G‐(β‐O‐4)‐G (dimers) and H‐(β‐O‐4)‐G‐(β‐O‐4)‐S (trimer). H‐ESI‐FT‐Orbitrap MS allows the qualitative characterization to obtain the Van Krevelen for the classes *Oy, Nx y Nx Oy* found in FPBO fractions (Entry 13). However, the complete chemical structures are still unknown. Among other techniques used to analyze LO and SLO, APPI with high‐resolution Orbitrap mass spectrometry stands out. This approach enabled the identification of a wide variety of LO from softwood and hardwood lignins, revealing signals of up to 3000 different oligomer species. These oligomers range from dimers to decamers, with mass ranges of 300–1800 Da (Entry 14).^[139]^

Despite the strengths of advanced mass spectrometry techniques, several challenges remain in identifying LO due to their heterogeneity. Many of these structures may have very similar mass‐to‐charge ratios, making them difficult to distinguish.^[140]^ Additionally, the high degree of fragmentation during ionization can complicate the interpretation of MS spectra.^[125]^ This fragmentation can obscure the identification of intact oligomers, further complicating the analysis.

An essential approach to addressing these challenges is the use of SLO. These model compounds provide well‐defined structures to help understand lignin‘s fragmentation patterns and ionization behaviors.[^137^, ^138^] By studying SLO, researchers can develop more accurate analytical methods and improve the interpretation of complex MS from LO mixtures. This strategy aids in benchmarking the analytical techniques and refining the protocols for more reliable characterization of LO.

### 2.2 Chromatographic Techniques

#### 2.2.1 SEC/GPC

Size exclusion chromatography (SEC) and/or GPC is a liquid chromatography technique that uses a column or series of columns (stationary phase) packed with a porous material to separate molecules based on their hydrodynamic volume. This technique can be used to isolate polar and nonpolar target fractions. Smaller compounds require more time to elute with the solvent (mobile phase) because they diffuse into the smaller pore network of the stationary phase. On the other hand, larger compounds elute faster since they spend less time in the porous areas.^[142]^ In this way, it is possible to separate compounds according to their size or weight‐average molecular weight (*M_W_*) and number‐average molecular weight (*M_W_*).

Several detector options or detector combinations are available to modify a chromatography device. While refractive index (RI) detectors are the standard, it is also common to employ ultraviolet detectors: Ultraviolet (UV), UV‐vis, diode array – DaD/PDA (190 – 900 nm), differential refractive index – RID (range of the instrument is usually 7×10^−9^ to 5×10^−4^ refractive index units full scale (RIU‐FS mode),^[143]^ viscometers, light scattering detectors (low angle, LALS; dual angle, DaLS; multi‐angle, MALS).^[142]^ MALS provides absolute molecular weight measurements without reference standards.^[144]^ This is particularly beneficial when working with unknown or complex samples such as LO, where accurate molecular weight determination can be challenging.

For SEC/GPC, it is necessary to perform a good calibration to guarantee the reliability of the results; the large hydrodynamic volume between the sample of lignin oligomers and commercially available calibration standards can also affect the quality of the molar masses determined by GPC.^[145]^ In addition, the separation is strongly affected by non‐SEC interactions, such as hydrophobic, electrostatic, and specific sorption effects. Non‐SEC interactions can be minimized by adding electrolytes and organic modifiers to the mobile phase and by increasing the column temperature.^[146]^ The preference in lignin analysis should be given to nonpolar stationary phase materials, which are not prone to hydrogen bonding, and using at least two sets of structurally different standards to achieve a predominant effect of particle size on resolution compared to pore size in GPC.^[147]^ However, lignin fragments from FPBO and BC involve polar and unipolar regions without further treatment as acetylation. Therefore, they are soluble in Dimethyl Sulfoxide (DMSO), and mixed columns are highlighted in Schuler et al.^[21]^ To our knowledge, only porous polyhydroxy methacrylate columns (Viscotek A‐Columns) are applicable for DMSO. They are designed by SEC to separate water‐soluble polymers and oligomers.

The literature shows various sample preparation methodologies and analytical conditions because there are still no established standard methods for this type of product.^[142]^

For FPBO from wood and solvent‐separated macro‐families (Entry 1 in Table 3), Dimethylformamide (DMF) was recommended for water‐soluble and water‐insoluble/CH_2_Cl_2_‐insoluble fractions for polyethylene glycol standards because tetrahydrofuran (THF) can change the solubility of the oils and affect their interactions with the columns. At the same time, THF is better for the analysis of toluene‐soluble and water‐insoluble/CH_2_Cl_2_‐soluble fractions and polystyrene standards because DMF may induce significant adsorption of non‐polar compounds.^[148]^ Higher Mw values were found than those of the compounds identified in the FPBO (Entry 2), (levoglucosan, cellobiosan, and 4‐allyl‐2,6‐dimethoxyphenol), suggesting that the unidentified species (sugars and pyrolytic lignin) are composed of heavy oligomers as dimers and trimers.^[149]^ In the same way (Entry 3), large fractions of high molecular weight compounds have been found, which were not requested to be identified by GC‐MS.^[150]^ Lower molecular weight molecules are identified in FPBO than in BC (Entry 4), because the latter has a higher concentration of vacuum residue (*M_w_* >1000 Da) than FPBO.[^151^, ^152^]

**Table 3:** SEC/GPC separation of oligomeric molecules of FPBOs and BCs from the thermochemical process.

Entry	Sample and solvent	Column	Calibration standard	Detection	*M_W_* (or *M_N_)* [Da]	Ref.
1	FPBO and fractions in DMF and THF	Shodex	polyethylene glycol polystyrenes	IR	Nonpolars: 600–2000 Polars: ~600 Volatiles: ~100 Monolignols: ~200 Extractives and Sugars: ~400	[148]
2	FPBO in THF	Shodex	Polystyrenes	DAD/RID	PL: 388 and 396 PDI: 1.84 and 1.81	[149]
3	FPBO organic oil fractions in THF	3 columns packed with polystyrene‐divinylbenzene	Polystyrenes	DAD (254 nm)	FPBO: ∼600–1600 PL isolated: ∼1000–2500	[150]
4	FPBO of FP, BC, and its fractions in THF	Preparative scale: three columns in series (Varian, PLgelMIXED‐bed E)	Polystyrene	VWD/RID (254 nm)	FPBO: <2000 HBO: <5000 (WL and GWL) and <9000 (GL)	[151,152]
5	BC (from black liquor) *	Styragel HR1 www‐sciencedirect‐com.ezproxy.unal.edu.co/topics/agricultural‐and‐biological‐sciences/polystyrene	Polystyrene	UV (270 nm)	Continuous flow: *M_W_*: 1089, *M_N_*: 672, PDI: 1.62 Batch: *M_W_*: 1348, *M_N_*: 805, PDI: 1.67	[160]
6	BC from Black liquor in DMSO	Viscothek Malvern A 2500 Porous poly hydroxymethacrylate polymer	Poly(styrene sulfonate) sodium salt	DAD (280 nm)	HBO (300 °C, 20 min) *M_N_*:~1839 and *M_W_*: 3727 Da, PDI: ~2.0 Lignin in Black Liquor M_N_:~6593 Da and Mw: ~8417 Da, PDI:~1.3 *(Data extracted from Figures)*	[141]
7	PL, PL‐FPBO, and fractions in THF	SS polymer – SDV	Polystyrene	MALS/RID (658 nm)	**FPBO**: *M_N_*: ∼580, *M_W_*: ∼980, PDI: 1.7, PL:7.2 %. **F1**: *M_N_*: ∼890, *M_W_*: ∼1490, PDI: 1.7, PL:16,1 %. **PL‐F1**: *M_N_*: ∼1100, *M_W_*: ∼1530, PDI: 1.4, PL:54,1 %. **PL‐FPBO**: *M_N_*: ∼1190, *M_W_*: ∼1640, PDI: 1.4, PL:54,1 %.	[154]
8	FP and CFP FPBO in THF	Polystyrene‐divinyl‐benzene copolymer gel beads (10^4^, 10^3^, and 50 Å)	Polystyrene	RI‐UV‐DAD (270 nm) dRI‐MALS (785 nm)	**FP**: *M_N_*: ∼300, M_W_: ∼500, 44 % FP (>500 g⋅mol^−1^) **CFP**: *M_N_*: ∼200, *M_W_*: ∼300, 44 % CPF (>500 g⋅mol^−1^). **MALS**: M_N_: ∼800, M_W_: ∼1500 (FP and CFP)	[157]

**GWL**: liquefaction in the guaiacol/water mixture, **WL**: liquefaction in water, **GL**: liquefaction in guaiacol, **BO**: Bio‐oils, **PL**: Pyrolytic lignin, **PL‐BO**: pyrolytic lignin fraction of bio‐oils, Variable wavelength detector (VWD), **CFP**: Catalytic fast pyrolysis, *Solvent not shown.

(Entries 5–6) SEC with BC from black liquor is applicable with the solvent DMSO and sufficient for an M_w_ range between 246 and 20700 Da, depending on the reaction conditions with the help of UV‐DAV detection 280 nm. Other non‐ionic samples can be analyzed using deionized water. For ionic and non‐ionic hydrophilic samples, salt solutions or buffer solutions can be used; for non‐ionic and ionic hydrophobic samples, adding polar organic solvents to the eluent is recommended. It also used mixed columns since lignin fragments have polar and nonpolar regions.

Using a combination of detectors can improve the quality and reliability of the results; i. e., laser and multiangle light scattering detectors (MALS/MALLS) can determine the absolute molar mass and the size of molecules (particles); therefore, it is possible to infer structural information, such as molecular branching, conformation, interactions, and aggregation. Thus, it is highly recommended that MALLS detectors be used as complementary to IR and UV.^[153]^ Another advantage of the SEC‐MALS‐ RID method is the possibility of quantifying PL fractions directly in the FPBO sample, whenever the *dn/dc* value of the sample is precisely known by integrating the DRI profile (Entry 7). However, since FPBO is a heterogeneous product (different molecules and a wide variety of chemical functionalities), to avoid errors caused by chemical heterogeneity, integration ranges can be established instead of a single value of *dn/dc* and calculated the percentages of fractions by weight of FPBO wet base.^[154]^ For one case (Entry 8), RI and UV‐DAD detectors indicate that FPBO has a higher *M_W_* and *M_N_* than catalytic fast pyrolysis oil (CFP) based on elution time. However, MALS determines that both oils have similar overall molecular weight distribution metrics (M_w_ and M_n_) and yield values significantly higher than those determined by RI and UV detectors.^[155]^ GPC, although limited in its ability to detect and analyze smaller, low molecular weight biopolymer‐derived monomers,[^153^, ^156^] plays a crucial role in identifying larger, higher molecular weight species such as recalcitrant oligomers derived from sugars and lignin, as well as polyaromatic and condensed products.^[157]^ It is important to note that comparing bio‐oil samples with varying abundances of UV‐absorbing species using UV‐DAD detection should only be done when the analysis specifically targets those species. Accurate determination of molecular weight values, mainly when using MALS, relies on consistent measurement of *dn/dc* across different oils. To advance the field, establishing a standardized analytical methodology for assessing the molecular weight distribution of FPBO is recommended. This methodology should encompass measurements of *dn/dc*, intrinsic viscosity, and molecular weight metrics relative to standards using GPC‐RI.^[157]^

The precise measurement of the molecular weight distribution of lignin oligomers remains unattainable owing to their intricate and heterogeneous characteristics. This is primarily due to the lack of standards for lignin oligomers, which are necessary for proper calibration. Until now, synthetic polymers, particularly polystyrene, have been predominantly used for calibration. However, polystyrene differs in its chemical structure from the molecular structures of lignin, which can introduce measurement errors. These differences in chemical structure can result in variations in how the polymers interact with the SEC/GPC stationary phase and detectors, consequently deviating from the expected calibration curve.[^17^, ^158^] While polystyrene is highly soluble in THF and DMSO, lignin is often insoluble or poorly soluble, leading to distorted chromatograms and incorrect molecular weight determination.^[17]^ Furthermore, polystyrene typically exhibits a random coil conformation in solution, whereas lignin oligomers can possess more branched or three‐dimensional structures. This disparity in molecular conformation can influence the exclusion or permeation of the analytes through the column, ultimately leading to inaccurate molecular weight determinations.^[159]^

To minimize measurement errors and enhance the characterization of LO using SEC/GPC, developing and utilizing standards designed explicitly for LOs is essential. These standards should closely match the LO chemical structure and molecular weight range to ensure precise calibration and reliable measurements. Further method development is also necessary, including optimizing column and solvent selection to improve separation and accuracy. Preparative‐scale GPC can separate fractions and facilitate in‐depth analysis using various techniques such as LC‐MS, NMR, GC‐MS, and FT‐ICR‐MS. This comprehensive approach allows for the determination of accurate molecular weights, molecular weight distribution, and identifying and quantifying the species present.

#### 2.2.2 Gas and liquid chromatography

Gas chromatography (GC) and liquid chromatography (LC) are separation techniques that, when coupled with various types of detectors, are useful for analyzing LO and SLO, which will be emphasized in this subsection. Table 4 shows the conditions and results of the analysis of LO and SLO by chromatographic techniques. These techniques can improve their efficiency when coupled to MS detectors, significantly improving their efficiency.

**Table 4:** Chromatographic elucidation applied for lignin oligomers.

Technique	Movil‐phase /Stationary phase	Standards	Compounds analyzed	Ref.
GC‐FID/MS	NS/HP‐5MS	Hexadecane as an internal standard	10 monomers quantified	[145]
GC–MS	NS/HP‐5MS	Not used	13 monomers identified and 5 quantified	[169]
GC×GC‐FID	NS/QAl_2_O_3_/Na2SO_4_ ‐ Mol Sieve 5 A	Internal standard: di‐n‐butyl ether (DBE)	7 Compound classes identify and quantified	[162]
GC×GC‐FID/MS	Helium/Mxt column (60 m) – ZB‐35HT (2.2 m)	Authentic monomers and similar structures of dimers	↑G monomers ↓S monomers 36 phenolic dimers and 21 trimers identified	[106]
UHPLC ‐ HRMSn/ PDA, LTQ Orbitrap	NS/BEH C18, BEH Phenyl, and CSH Phenyl‐Hexyl	Not used	36 tentative structures of oligomers	[140].
LC‐ESI‐MS/MS	Water:ACN gradient mode/Zorbax eclipse C18	Phenolic compounds and LO	G‐type LO: 3 dimers, 2 trimers 2 tetramers	[172]
UHPSFC/QTOF‐MS	scCO_2_/seven columns	40 lignin phenols	36 monomers identified and quantified	[173]

*NS: Not shown, ACN: Acetonitrile.

Gas chromatography‐Flame Ionization Detection (GC‐FID) and Gas Chromatography‐Mass Spectrometry (GC‐MS) and their two‐dimensional versions (GC×GC‐MS/FID) are efficient techniques for the separation and analysis of low‐weight molecular LO.[^161^, ^162^]

During GC, the sample is vaporized and carried by the mobile phase (carrier gas) through the stationary phase (column). The separation is based on the relative vapor pressure of the compound and its affinity for the stationary phase. Similar compounds tend to separate more effectively in similar stationary phases. Therefore, non‐polar analytes are best separated using non‐polar columns with dimethyl or a low percentage of diphenyl. Compounds capable of π‐π interactions can be separated using stationary phases containing phenyl groups. Acids and alcohols, capable of hydrogen bonding, are ideally separated using Polyethylene Glycol (PEG) columns unless they have been derivatized to reduce polarity^[163]^ as well as polyethylene glycol oligomers.^[164]^ However, GC is limited to volatile compounds with a molecular weight below 1250 Da and those that are thermally stable enough to avoid degradation before reaching the detectors (FID/MS).^[165]^

Therefore, low molecular weight monomers, dimers, and certain trimers obtained through the thermochemical depolymerization of lignin can be separated and analyzed using GC/MS‐FID and GC x GC/MS‐FID using a high‐temperature resistant column (tolerance up to 420 °C while generally columns operate at 260–350 °C) and usually, a derivatization step to increase its volatility,[^17^, ^145^, ^166^, ^167^, ^168^] but is not possible analyze bigger molecules. GC‐FID/MS facilitated the separation, identification, and quantification of monomers: Guaiacol, Ethyl Phenol, o‐Cresol, Ethyl Guaiacol, Syringol, Propyl Guaiacol, Methyl, Ethyl, and Propyl Syringol, and Acetosyringone in lignin **li**quefaction products.^[145]^ Compound classes: Alkylphenolics, Guaiacols, Catechols, Aromatics, Linear/branched alkanes, Cyclic ring alkanes, and Ketones/alcohols were identified in FPBOBC using GCxGC‐FID.^[162]^

Dao Thi et al.,^[167]^ analyzed six lignin oil fractions (reductive catalytic fractionation) using high‐temperature (40 to 420 °C) GCxGC‐FID/MS. The fractions were previously derivatized with N‐methyl‐N‐(trimethylsilyl) trifluoroacetamide to prevent interaction between the hydroxyl groups of the phenolic compounds and the column. More than 80 % of the structural molecular units within LO were unambiguously assigned, including β‐5 γ‐OH, β‐1 γ‐OH, β–β 2× γ‐OH, β‐5 ethyl, β‐1 ethyl, β–β, and 5–5 inter‐unit linkages. However, only 11 monomers were quantified by 1D GC‐MS using retention indices from authentic standards or the NIST library. Structures of 36 dimers, up to 648 Da, and 21 trimers, up to 974 Da, were assigned based on a detailed analysis of their mass fragmentation patterns. Similarly, by GC‐MS, phenolic monomers and oligomers extracted with diethyl ether (DEE) from BC of hardwood lignin were analyzed.^[169]^ However, the identification by retention index could not be applied to all dimers and trimers due to a lack of matches with the library and authentic standards.^[31]^ This is because these methods enable the identification of their chemical structures, which can be matched with available mass spectral databases, either open‐source (e. g., NIST 23)[^170^, ^171^] or commercial (e. g., Wiley MS registries). However, numerous LO still lack a match, resulting in unknown structures. Consequently, identifying these compounds relies solely on interpreting their mass spectra, which proves challenging due to the complex chemistry of oligomer fragmentation induced by ionization.

Complementary techniques such as SEC/GPC, FTIR, and NMR have been applied to comprehensively analyze the LO. Furthermore, the utilization of Liquid Chromatography‐Mass Spectrometry (LC‐MS), High‐Performance Liquid Chromatography – Diode Array Detection (HPLC‐DAD), or its combinations of HPLC‐MS and HPLC‐DAD‐MS can facilitate the separation and analysis of higher molecular weight LO. Liquid Chromatography‐Electrospray Ionization‐Tandem Mass Spectrometry (LC‐ESI‐MS/MS) effectively elucidates the type of linkages in the complex lignin structure. Using LC‐ESI‐MS/MS, LO and LO G‐type (with G units) were identified.^[172]^ This identification process involved making certain defragmentation assumptions and enhancing separation and ionization efficiency using mobile phase additives such as ammonium formate (AmF) or formic acid (FA). Consequently, unequivocal structural characterization of LO was achieved. However, the number of identified LO remains limited, and many others remain unknown,^[172]^ because, as in GC‐MS, the identification of these oligomers is hindered by the absence of several molecular ion profiles in MS libraries.^[17]^

One advantage of MS/MS or n‐MS over Pyrolysis‐Gas Chromatography‐Mass Spectrometry (Py‐GC‐MS) or NMR is the capability to investigate the chemical structure of LO through their MS fragmentation pathways. By combining liquid chromatography (LC) with high‐resolution mass analyzers, various identification methods for LO have been established. Utilizing a PCA‐QDA (Principal Component Analysis – Quadratic Discriminant Analysis) model and Kraft LOs analysis through UHPLC/HRMS^n^ (Ultra‐High‐Performance Liquid Chromatography coupled with High‐Resolution Multi‐Stage Mass Spectrometry), tentative structures are proposed for the 36 tentative LO (including dimers, trimers, and tetramers) that were achieved among 587 detected peaks.^[140]^ However, it should be noted that this approach requires specialized and costly equipment, and the identified structures remain tentative, potentially differing from the actual molecules due to the absence of standards.

Another chromatographic technique known as supercritical fluid chromatography (SFC), which utilizes supercritical scCO_2_ as the mobile phase in combination with reversed‐phase HPLC, has demonstrated efficiency in separating lignin dimers and oligomers. Using UHPSFC/QTOF‐MS (Ultra‐High‐Performance Supercritical Fluid Chromatography/Quadrupole Time‐of‐Flight Mass Spectrometry), 36 lignin‐derived monomers were separated, identified, and quantified in less than 7 min, showing selectivity and separation speed advantages over other chromatographic techniques.^[173]^

The absence of well‐characterized LO standards hinders the calibration of mass spectrometers and a good characterization of the chemical structures of the LO present in the liquid products of the derivatization of lignin and lignocellulose. SLO standards or model compounds with known chemical structures serve as valuable reference compounds for the structural elucidation of LO. These model compounds can provide information on fragmentation patterns, enclosures, and precise size. Furthermore, these SLO can be used as standards for quantitative purposes since they allow for determining the concentration or abundance of LO types in fractionated FPBO and BC samples, developing and optimizing new specialized analytical methods, their validation, and reproducibility. Therefore, it is recommended that future research focus on obtaining SLO to be used as model compounds or standards, along with developing isolation and purification strategies.

Therefore, to facilitate a more comprehensive qualitative and quantitative analysis, it is evident that the development of viable SLO is required to establish a mass spectral (MS) library. Considering that the number of unknowns LO is enormous, obtaining every one of the corresponding standards would be impossible. Therefore, synthetically producing model molecules (SLO) with similar key chemical structures would be a decisive fundamental step. The knowledge of their mass spectra can be attested to how their ionization‐induced defragmentation occurs, acting as a fingerprint. By determining similar LO that generate coincidences in three or more main ions, it would be possible to identify and classify the chemical structures of the analytes into groups or classes of compounds with some similarities.

Overcoming these limitations requires big efforts to synthesize and characterize SLO that are very similar in complexity and diversity to the LO, which is why, in addition to laboratory work, the use of computational tools and DFT is required that will allow predicting theoretical MS spectra based on experimental SLO. To begin, efforts should be directed towards identifying additional dimers and trimers through mass spectral analysis using GC×GC FID/MS in combination with NMR,^[106]^ for bigger molecules LC–MS, Orbitrap LC–MS, MALDI‐TOF‐MS.[^17^, ^127^, ^174^] This comprehensive approach will improve results and help us better understand LO. LO standards can be obtained by isolating them from natural lignin sources or synthesizing them from monomers, as described in section 3.1.1.

Chromatographic and spectroscopic analytical techniques, as well as their combinations, are inherently limited by the diversity, molecular size, and structural stability of LO. Selecting the most appropriate technique is a complex task that requires careful consideration of multiple factors, including molecular weight, volatility, thermal stability, and the availability of reference standards. Table 5 summarizes the key analytical techniques used for LO characterization, detailing their suitability, limitations, and the type of structural information they provide.

**Table 5:** Summary of key analytical techniques used for LO characterization.

Type of LO	Technique	Suitability	Limitations	Characteristics
<500 Da	GC‐MS/FID^[162]^	Monomers and small dimers after derivatization	Still limited by volatility and thermal stability	Complete structural information (MS spectrum)
Up to ~1200 Da,	GCxGC‐MS/FID^[106]^	Dimers, trimers and some tetramers	Compounds must be volatile or derivatized.	Complete structural information (MS spectrum)
150 – ~700 Da	FT‐ICR MS	Monomers to trimers (Technique capable of analysing organic compounds between 150 −1500 Da^[195]^, although LO up to 1500 Da has not been reported yet)	Low‐ionization efficiency for some lignin fractions, especially high Mw oligomers, may cause underrepresentation of certain compounds.	Functional groups, units, Exact mass, molecular Formula, linkages, Aromaticity index (AI), etc.
From monomers to larger LO	HPLC coupled to UV, DAD, or ELSD.	Recommended for large, non‐volatile and thermolabile LO	Require standards	Identify structures by retention time (comparative technique)
From monomers to larger LO	LC‐MS	Recommended for large, non‐volatile and thermolabile LO	Require database	Characterization based on molecular weight and fragmentation patterns
From monomers to larger LO	2D NMR (HSQC, HMBC, ROESY, NOESY)^[109]^	For quick identification of LO type of any size Confirmation of units and linkages	Requires relatively pure samples for accurate interpretation. Limited to soluble lignin fractions	Provides semi‐quantitative data on the relative abundance of different lignin linkages and units.
Up to 3000 Da	Orbitrap LC–MS	Wood lignin dimers – decamers^[196]^	Expensive, complex data processing, requires spectral databases	Exact mass, units, linkages, and isomer differentiation.

Selecting the most appropriate technique is a complex process that requires careful evaluation of multiple factors, including molecular weight, volatility, thermal stability, and the availability of reference standards.

### 2.3 Techniques for Thermophysical Characterization

Knowledge of the thermal degradation behavior of materials is a very relevant parameter, providing valuable information on the behavior of substances, stability, and glimpses of possible applications. Determining the thermal stability of substances (LO, SLO) is crucial to understand their performance at different temperatures, for which thermogravimetric analysis (TGA) is used, which measures weight loss as a function of temperature and infers degradation patterns and kinetics.[^9^, ^175^, ^176^] Analysis by Differential Scanning Calorimetry (DSC) and Modulated Differential Scanning Calorimetry (MDSC) can measure thermophysical properties (temperature and phase transition enthalpy), such as the glass transition (*T*_*g*,_ *ΔH_g_*), melting (*T*_*m*,_ *ΔH_m_*), crystallization (*T*_*cr*,_ *ΔH_cr_*), and heat capacity (*C_p_*).[^117^, ^177^] DSC is also a useful technique for studying synthetic and bio‐based polymers′ reactivity and decomposition pathway,^[178]^ kinetics, and determining types of endothermic or exothermic reactions.^[179]^ Furthermore, by DSC it is possible to determine the purity of a substance based on van′t Hoff′s law of depression of the melting point of eutectic systems by the standard method ASTM E928‐19.^[180]^ This method is frequently available in the equipment software and only requires knowing the melting point of the pure reference substance, it is widely applied in the analysis of substances for pharmaceutical and material sciences,[^181^, ^182^] as well as measuring the purity of several bio‐based molecules.[^183^, ^184^]

#### 2.3.1 Estimation of Thermophysical Properties

Experimental data is virtually always missing when developing novel or model molecules, such as LO or SLO. Estimating thermophysical properties for these species when required, such as process modeling or comparison (e. g., phase equilibrium and particle models) is necessary.^[185]^ Fonseca and Funke^[186]^ reviewed the methods available for the estimation of these parameters, focusing on Quantitative Structure‐Property Relationship (QSPR) models, namely group contribution methods, which enjoy wide use due to ease of use and accuracy.^[187]^ Other methods include DFT and Artificial Neural Network (ANN) models, as well as solutions making use of all three paradigms (QSPR+DFT+ANN), which permit the estimation of these properties based on quantum chemical theory and machine learning, respectively, and enjoy increasing attention and accuracy.[^188^, ^189^, ^190^]

There are frequently multiple options to estimate a single property, and it ultimately falls upon each user to select the most reliable method and value.^[191]^ Several software tools are available for easy estimation of some of these parameters, such as flowsheeting software (e. g., Aspen Properties™ within Aspen Plus™ or HYSYS™) or property estimation engines (e. g., NIST ThermoData Engine, ACD/Labs Percepta™). Fonts et al.^[192]^ used some of these methods to estimate properties for one pyrolytic lignin surrogate and one hybrid oligomer of lignin and sugar moieties in the context of modeling FPBO. Manrique et al.^[193]^ estimated some properties of the LO they proposed. Gorensek et al.,^[194]^ devised alternative QSPR‐like strategies to estimate several of these properties for lignocellulosic reaction surrogates, including lignin representatives and FPBO components.

A complete characterization of LO and SLO is crucial to identify or develop new applications for these molecules and enhance the valorization of lignin. Modeling any chemical or thermochemical process or separation process requires a thorough understanding of the substances involved, including their thermodynamic properties, physical characteristics, and well‐defined chemical structures. Next, in Section 3, the structures of SLO that are known up to now will be described. Thermophysical data has been estimated for several of these structures, and the values can be found in the Supplementary Information.

## 3 Synthesis of Lignin Oligomers

Synthetic lignin oligomers (SLO) with well‐defined structures are used to study the breakdown pathways of lignin during depolymerization processes, such as thermochemical, enzymatic, or fractionation methods.^[197]^ By analyzing the decomposition of SLO, researchers can identify potential products and optimize process conditions, as well as discover new catalysts and solvents.^[17]^ Additionally, SLO can be used as model molecules or standards to calibrate analytical techniques, such as chromatography or spectrophotometry. This enables the comparison of signals from lignin oil samples, facilitating separation and purification processes based on their similar properties. Furthermore, SLO can aid in creating databases for characterizing various lignin oils and their depolymerization products, ultimately enhancing the overall analysis of LO.

It is important to note that functional monomers, which can streamline the development of renewable polymeric materials with distinct properties, could also be considered SLO in their own right. These materials have the potential to replace fossil‐based counterparts, such as porous polymers based on divanillin (DPP) and 2,5‐furandicarboxaldehyde (FPP).^[198]^ They find applications in contaminant absorption, UV‐resistant polymers, adhesives, and medical uses,^[199]^ drug delivers,^[200]^ fibers and fabrics,^[201]^ and antimicrobials.^[202]^ These advancements are pivotal in enhancing the efficiency of processes and products within emerging biorefineries. In the following, different possibilities for the production of SLO is summarized and discussed in detail (section 3.1). Purification of SLO is of decisive importance for their application as standards and will be covered in a separate section (section 3.2).

### 3.1 Production of Synthetic Lignin Oligomers

SLO can be obtained through various methods, including the synthesis of modified lignin derivatives or the selective synthesis from monomers.^[203]^ Depolymerization methods break down natural lignin into smaller units, which can then be used as building blocks for synthesizing oligomers or modified structures. Monomers can undergo various polymerization reactions, such as radical polymerization, condensation polymerization, or enzymatic polymerization, to form lignin‐like oligomers.^[204]^ Functionalizing LO through chemical and enzymatic routes is a key approach, employing techniques such as hydroxymethylation, aminomethylation, nitration, sulfomethylation, and sulfonation (see Table 6). These modifications enhance their solubility and reactivity, resulting in chemical structures that are more suitable for various applications, including polymeric resins, adhesives, and coatings.^[205]^

**Table 6:** Synthetic Lignin Oligomers: advantages and limitations of LO modification methods.

Reactions Involved	Conditions	Functional groups	Selectivity	Advantages	Limitations	Ref.
Synthesis of modified lignin derivatives						
Esterification: Lignin with carboxylic acids or acid derivatives: – Acyl chlorides, – Carboxylic anhydrides, – Carboxylic acids, and – Lactones	Combined with other stabilization strategies	Esters Aliphatic hydroxyl	Esterification of α and γ positions of the β‐O‐4′ motifs	Obtain functionalized lignin derivatives with desirable properties for new applications: – Green composite materials – Improve the compatibility – Improved hydrophobicity – Others	– The availability and cost of suitable esterification reagents limit the scalability	[207,208]
	Catalyst‐free esterification (160 °C for 24 h)	Aliphatic OH	Modified lignin of various carbon lengths for applications such as biobased polyesters or hydrophobic coatings	– A green procedure with low environmental factor (E‐factor) – Modify the aliphatic OH group without causing degradation and repolymerization	No identified	[209]
Etherification: Introduction of ether linkages into the lignin structure	Direct etherification catalyzed by Zr under mild conditions	Hydroxyl	Allylation of lignin and model compounds G‐G′ β‐O‐4′ ether type. Selectively target the benzylic hydroxyl functionalities	– Kinetic analysis was carried out. – Expanding its chemical versatility of molecules – Improving thermal stability, mechanical strength, and hydrophobicity.	– Some structural alterations – Complexity of lignin, reaction kinetics, mass transfer limitations, and separation processes limit the scalability	[210]
Acylation: Catalyzed reaction between a suitable acylating agent and the hydroxyl	Acylation in two stages	Benzylic	Good benzylic carbon (Cα) selectivity over Cγ of lignin	– The acyl groups can impart increased solubility, compatibility, and chemical reactivity.	– Limitations could arise from factors such as sterical hindrance – In general lack of Selectivity – Structural Integrity – Scalability and cost limitations	[211,212]
Methylation: Catalyzed reaction between lignin and a methylating agent	Methylation of model compounds and hydroxymethylation	Phenols Aliphatic Hydroxyl	Eudesmin/yangambin structures: – Pinoresinol – Eudesmi Hydroxymethylation increases aliphatic hydroxyl group content	– Increased Hydrophobicity – Stability Enhancement	– Achieving selectivity is still a challenge – Possible structural alterations – Scalability and cost limitations	[25,208,213]
Aminomethylation: Amino groups are introduced using formaldehyde and amines	Mannich reaction under alkaline, neutral, or acidic conditions	Amino (−NH_2_) groups	Incorporating amino groups primarily at the ortho or para positions relative to the hydroxyl groups.	Structures with potential applications as cationic surfactants	May require additional purification steps to achieve desired product purity	[214]
*Nitration*: Treated with nitrating agents	Nitrating agents in non‐aqueous solvents like acetic anhydride and acetic acid.	Nitro (−NO_2_) groups	It targets aromatic rings, particularly at the ortho and para positions relative to hydroxyl groups.	Potential for producing electrocatalyst precursors for oxygen reduction reactions.	The reactions are typically hazardous and require careful handling.	[205]
*Sulfomethylation*: Incorporation of methylene sulfonate groups.	Reaction with formaldehyde and an alkali metal sulfite salt under alkaline conditions	Methylene sulfonate groups	It targets phenolic hydroxyl groups, introducing sulfonate groups at the benzylic positions.	For development of adsorption materials for cationic dyes.	Requires strict control of reaction conditions	[206]
*Sulfonation*: Direct addition of sulfonate groups	Treatment with sulfuric acid or sodium sulfite.	Sulfonate groups	Formation of covalent carbon‐sulfur bonds	Formation of lignosulfonates used as dispersants and binders	May lead to degradation of the lignin structure	[206,214]
*Demethylation*: Conversion of methoxy groups to phenolic hydroxyl group.	Typically performed under acidic conditions.	Phenolic hydroxyl (−OH) groups	It targets methoxy groups attached to the aromatic rings, converting them into hydroxyl groups.	Enhances lignin‘s reactivity, making it a better precursor for resins and adhesives.	Requires careful handling due to the corrosive nature of reagents.	[215]
Polymerization						
Radical polymerization: Formation of radical species from initiators and polymerization by the radicals	Reversible addition‐fragmentation chain transfer (RAFT) and free radical polymerization (FRP)	Combinatorial radical coupling process	Methacrylate polymers with different thermal and viscoelastic properties via RAFT Homopolymers and block copolymers synthesis via RAFT Divinyl compound from vanillin Lignin‐g‐poly‐NIPAM copolymers	Versatility to allow the synthesis of a wide range of lignin oligomers A promising alternative for the synthesis of polymers to replace petroleum‐derived monomers with renewable monomer precursor Viability to scale up	Possible formation of a mixture of oligomers with varying sizes and structures Starting from monofunctional monomers (i. e., individual hydroxyl molecules) only linear thermoplastic polymers are produced Possible degradation or side reactions Purifying lignin oligomers synthesized via radical polymerization can be challenging	[216,217]
Condensation: Formation of covalent bonds between the lignin units to form bigger chains	Catalyzed condensation at 200–220 °C	Schiff base bonds by a condensation reaction of an aldehyde and an amine. The aldehyde group of vanillin could be used to react with an amine group.	– Vanillyl alcohol‐based polymers – Polymers derived from vanillyl alcohol, syringaldehyde, eugenol, and ferulic acid: Polyimines‐polybenzoxyazines	Enable the synthesis of well‐defined lignin oligomers Produce relatively pure lignin oligomers SLO with good compatibility with other polymers or materials	Potentially leading to undesired side reactions or inconsistent results Formation of higher molecular weight lignin oligomers difficult to separate reproducibility can be challenging due to the complexity of lignin as a starting material.	[^218^, ^219^]

Lignin‐based polymers and hydrogels show great promise for a range of applications. LO can be used to produce polymeric resins with better mechanical and thermal properties compared to traditional petrochemical resins. Hydrogels, which can be customized for uses such as drug delivery, wound healing, and environmental remediation, represent another important area of development.[^205^, ^206^] Although these structures are not fully synthetic, they can be considered as such due to the extensive chemical modifications made to their original structure.

#### 3.1.1 Design of SLO standards based on common linkages

**Figure 6:**
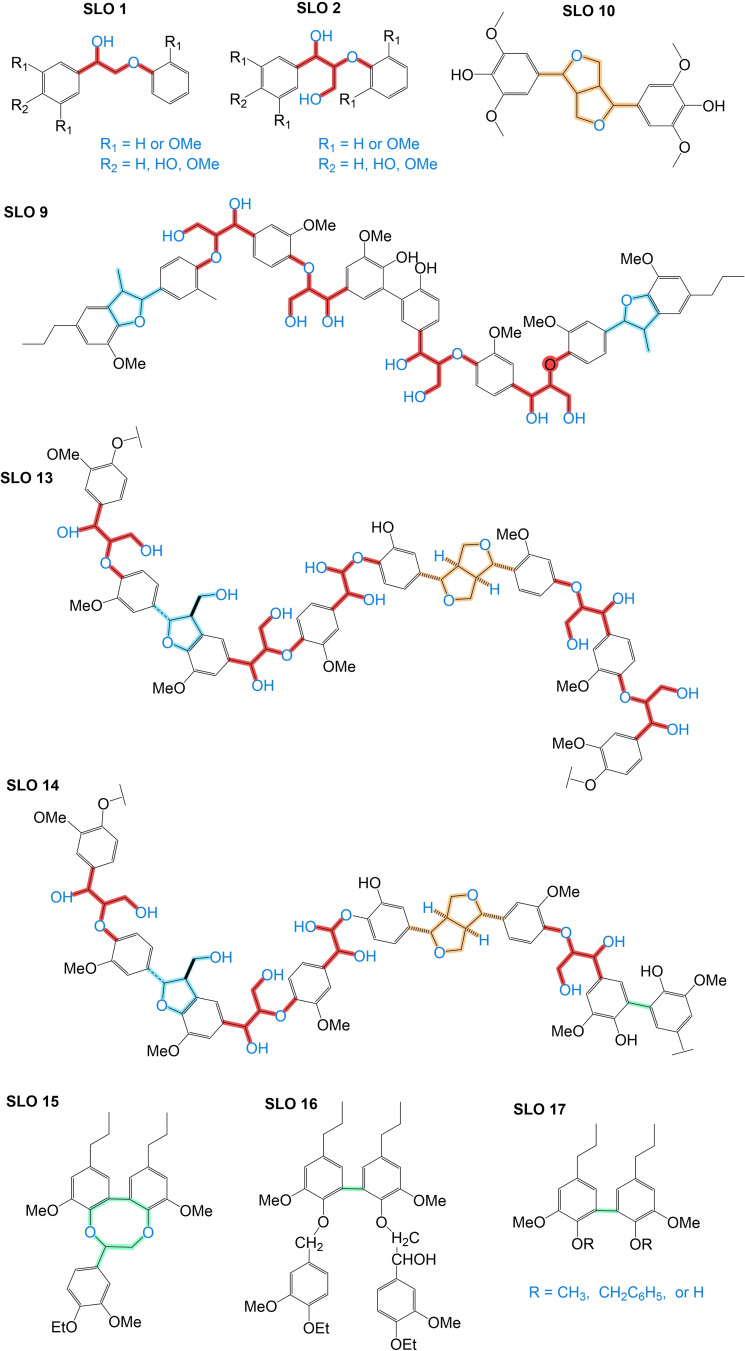
Molecular structures of the SLOs obtained by chemical and enzymatic synthesis referenced in Entries 1, 4, 5, 6, and 7 (Table 6). SLO 1–SLO 2 adapted from Ref. [25] Copyright (2020) with permission from Wiley. SLO 9 adapted from Ref. [224] Copyright (2013) with permission from RSC. SLO 13–14 adapted from Ref. [105] Copyright (2015) with permission from RSC. SLO 15–17 adapted from Ref. [225] Copyright (2004) with permission from Elsevier.

**Scheme 1:**
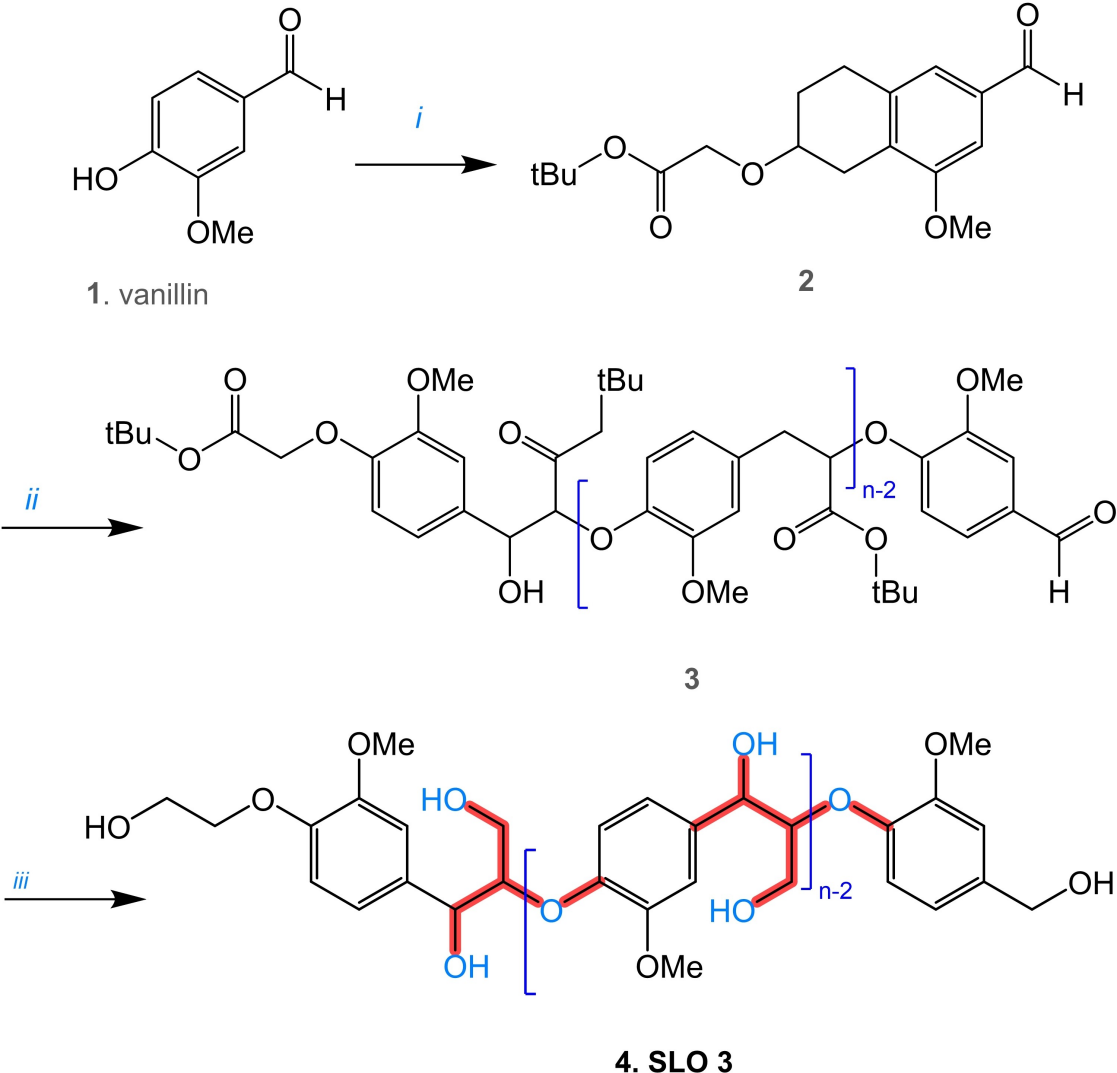
Mechanisms for the synthesis method of Katahira et al.,^[23]^ with slight modifications. Adapted from Ref.^[109]^ Copyright (2021), with permission from De Gruyter.

**Scheme 2:**
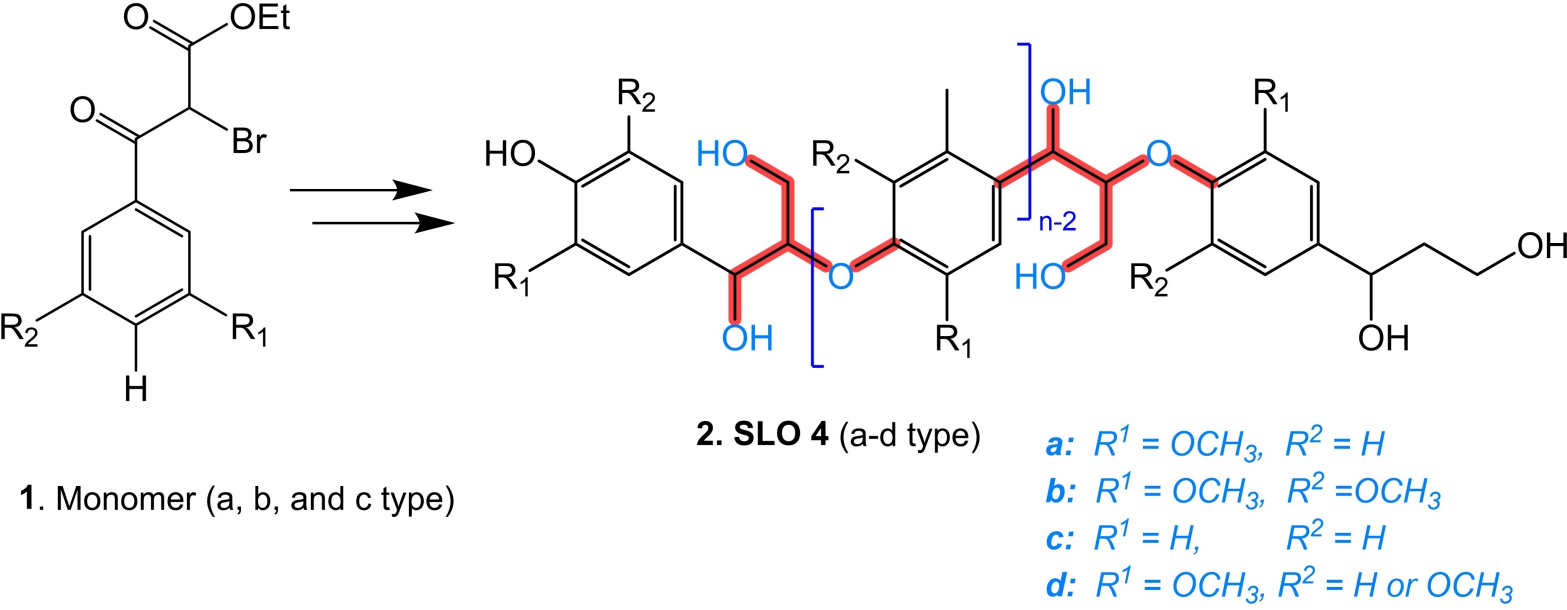
Mechanisms for the synthesis method of Kishimoto et al.[^103^, ^131^] Adapted from Ref. [131] Copyright (2008), with permission from RSC.

**Scheme 3:**
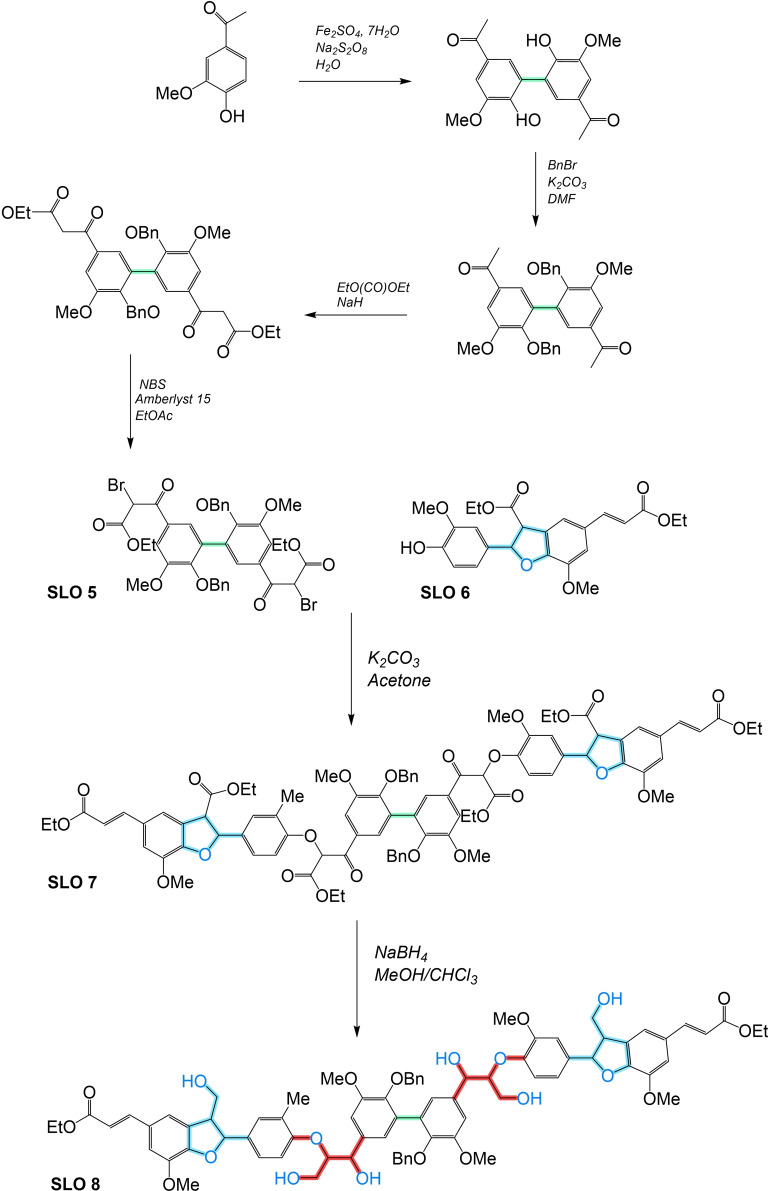
Mechanisms for the synthesis method of Graham et al. Adapted from Ref. [224] Copyright (2013) with permission from RSC.

**Scheme 4:**
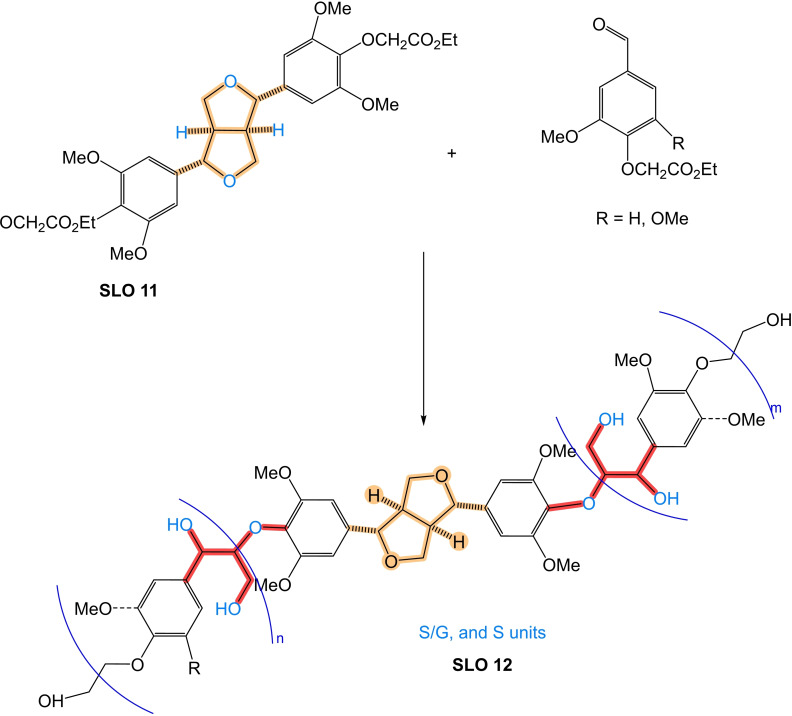
Mechanisms for the synthesis method of Lancefield and Westwood. Adapted from Ref. [105] Copyright (2015), with permission from RSC.

**Scheme 5:**
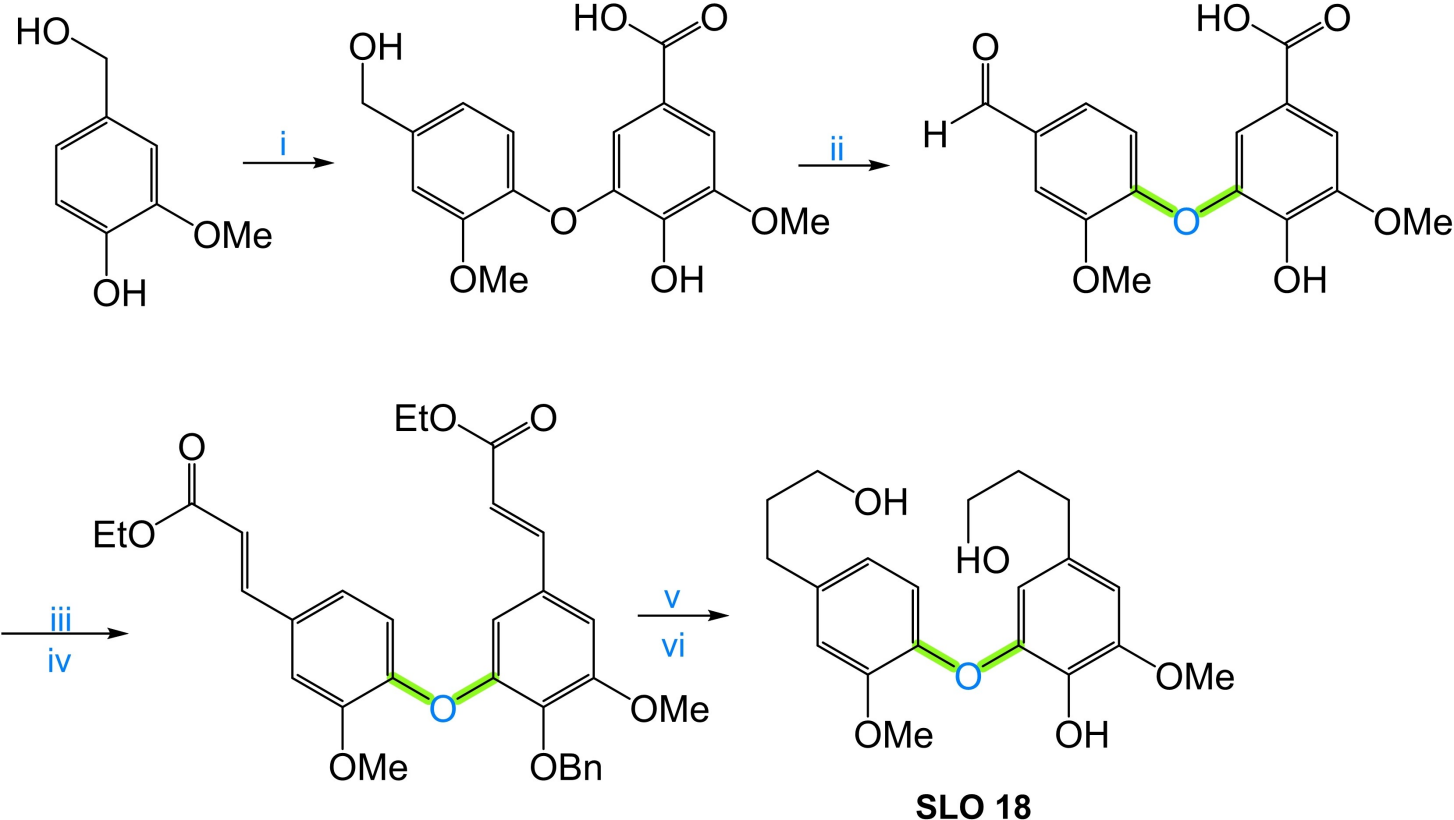
Mechanisms for the synthesis method of Li et al. Adapted from Ref. [230] Copyright (2020), with permission from Wiley.

**Scheme 6:**
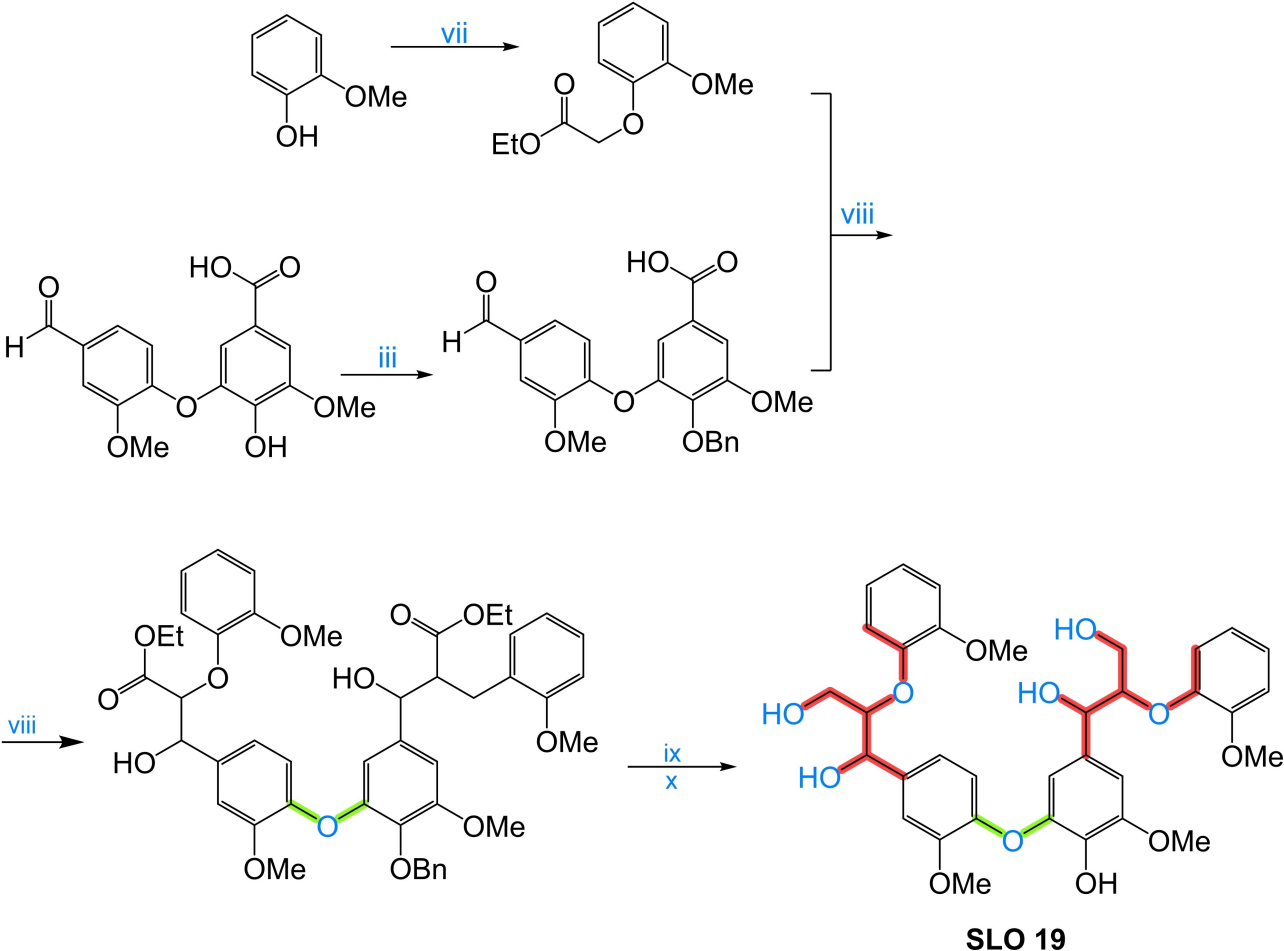
Mechanisms for the synthesis method of Li et al. Adapted from Ref. [230] Copyright (2020), with permission from Wiley.

This section focuses on synthesizing chemical structures to be used as models/standards using the most relevant linkages present in lignin. These can be used as model molecules to study thermochemical conversion reactions or as calibration standards for analytics. Figure 6 illustrates several chemical structures that can be synthesized from monomers using chemical and enzymatic methods. These structures, along with key recommendations and considerations, are summarized in Table 7. Schemes 1, 2, 3, 4, 5, 6 detail the steps and the network of reactions necessary to build some of the SLO.

**Table 7:** Lignin oligomer linkages were obtained by synthetic methods, as well as main reactions and recommendations.

Entry	Reactions	Target‐specific compound	Recommendations	Method
1	Williamson ether synthesis, bromination, substitution, condensation, and reduction.	SLO 1 type B (Figure 6): Dimer β‐O‐4 C_6‐_C_2_ compounds (C_6_ of the aromatic ring and the C_2_ of the ethyl chain). SLO 2 Type C (Figure 6): Dimer β‐O‐4 C_6_‐C_3_ compounds (C_6_‐C_3_ β‐O‐4 and γ‐carbinol group (‐CH_2_OH))	B can be recrystallized with good yield. C involves several steps and moderate yield results and are more representative models of the β‐O‐4 linking motif. Adler′s method requires less specialized equipment but some expensive reagents.	Adler, 1955^[220]^ Nakatsubo, (1975)^[221]^ cited by Lahive et al. (2020)^[25]^, Buendia et al. (2011)^[231]^, Dabral et al. (2018)^[223]^.
2	Synthesis of t‐butocarbonylmethyl vanillin 1, oligomerization 2, and reduction 3 as Mechanism in Scheme 1.	SLO 3: Oligomer β‐O‐4 composed only of erythroisomers with a short chain: 613 Da and a long chain: 809 Da.	Feasible to eValuate for other dimers as co‐oligomers containing G, S, and H may be created by use of a mixture of the corresponding starting monomers. Use CC to separate the products.	Katahira, 2006 with slight modifications.[^23^, ^109^]
3	Polycondensation of a brominated monomer 1 by Williamson ether formation and subsequent reduction of the carbonyl polymer 2 (Scheme 2)	SLO 4: Trimers β‐O‐4 a: G with R1=OCH_3_, R2=H (Mw:5460 Da) b: S with R1=OCH_3_, R2=OCH3 (Mw: 5950 Da) c: H with R1=H, R2=H (9650 Da) d: G/S with R1=OCH_3_, R2 =H or OCH_3_ (Mw: 4120 Da)	Polymerization using potassium carbonate in anhydrous DMF at rt for 24 h to afford polymers1b–1d with yields of 88 %, 97 %, and 90 %, respectively. The crude products were purified by precipitation in ether, to remove the low molecular weight compounds, to afford polymers2b–2d with yields of 27 %, 66 %, and 18 %, respectively.	Kishimoto et al. (2008).[^103^, ^131^]
4	Oxidative phenol coupling, bromination, polymerization, reduction, and condensation reactions (Scheme 3)	SLO 5: symmetrically dibrominated molecule SLO 6 and 7: intermediate dimer and hexamer also useful as lignin models SLO 8: hexamer β‐5, β‐O‐4 and lignin‐ferulate crosslinks SLO 9: octamer β‐5, β‐O‐4 linkages, and lack functionality on the propyl side chains.1	SLO5: yield 79 % using Amberlyst‐15® acidic resin and N‐bromosuccinimide in ethyl acetate. SLO8: yield >8.45 % starting from SLO5 (building block) SLO 9 : 7 % overall linear yield in seven steps.	Graham et al. (2013)^[224]^
5	Radical‐radical coupling for β‐β formation and dismutation for β‐O‐4 linkage.	Production of a mixture of 2,6‐DM A, β‐β and β‐O‐4 dimers from sinapyl alcohol, which can be favored to dimer β‐β syringaresinol (Figure 6 **SLO 10**) of high purity.	Chemical: yield until 67 % of syringaresinol Enzymatic: multigram scale (93 % yield) of syringaresinol which is a commercial standard. Enzymatic method has kinetic parameters	Tran et al. (2015)^[232]^ Jaufurally et al. (2016)^[228]^
6	Chemical biomimetic dimerization and enzymatic dimerization (Scheme 4).	Facile synthesis of an S‐G β‐O‐4 and β–β as a model of hardwood lignin (SLO 12) tetramer from SLO 11. Softwood lignin model (SLO 13) G octamer with β‐O‐4, β–β and β‐5 units phenolic softwood model (SLO 14) G octamer with 5–5 phenolic groups	SLO11 doesn′t need CC for purification SLO12: 48 % yield over 2 steps on a 2 g scale SLO13: 40 % yield SLO14 with 32 % yield over 2 steps Feasible method for big SLO able to study lignin degradation	Lancefield and Westwood^[105]^
7	Chemical and enzymatic dimerization	Non‐phenolic dibenzodioxins lignin models (SLO 15 −17) with 5–5^*^, α‐O‐4 and β‐O‐4 units shows in Figure 6.	SLO 15–17 global yield starting from 5 is 34 % 50 % yield for dibenzodioxins‐1 and has been improved from 8 % to 53 % for dibenzodioxins‐2 using Ag_2_O	Gardrat et al. (2004)229 and Karhunen et al. (1995)^[229]^
8	Radical coupling reactions in 6 steps: Ag_2_O to promote oxidative dimerization of vanillyl alcohol, continuing with sidechain oxidation, olefination, and hydrogenation (Scheme 5).	Dimer 4‐O‐5 diaryl ether (SLO 18)	Compound model, bearing two hydroxypropyl sidechains	Li et al. (2020)^[230]^
	Starting from the aldehyde intermediate of step (ii), it was a precursor to two. trimeric and one tetramer models (Scheme 6).	Tetramer 4–0–5 (SLO 19)	In up to 10 steps, it is possible to obtain trimers and a model tetramer.	

*The properties of all SLO named in this table and the parameters used for their estimation are detailed in the Supporting Information.

According to Lahive et al.,^[25]^ commercial (2‐phenoxyethyl)benzene (type A) is used as an LO model but lacks both α and γ hydroxyl groups. So, Adler et al.^[220]^ and Nakatsubo et al.^[221]^ can obtain dimers β‐O‐4 Type B (SLO 1) incorporating the benzylic hydroxyl group at the α position, and β‐O‐4 Type C (SLO 2) adding the γ‐carbinol group, ‐CH_2_OH, to improve the representability of a natural oligomer. Various researchers have previously used these methods to obtain SLO and modify it by oxidation reactions (Entry 1).[^132^, ^222^, ^223^]

The synthesis of a model β‐O‐4 type oligomeric lignin from vanillin using the Katahira method[^23^, ^109^] (Figure 6 and Entry 2), involves a three‐step reaction process: (i) the synthesis of t‐butoxycarbonylmethyl vanillin (2), (ii) nucleophilic addition polymerization of the compound, and (iii) reduction of the oligomeric β‐hydroxyl ester as Scheme 1. Tokunaga et al.^[109]^ utilized this method to produce a trimer model, confirming the presence of a β‐O‐4 bond and establishing that its structures solely consist of erythro‐isomers through NMR analysis, as was detailed in Section 2 (Table 2 *, Entry 6*). This method is highlighted because using the corresponding base monomers can also be employed to obtain other lignin structures, such as Syringyl and p‐hydroxyphenyl nuclei. Using the Kishimoto method (Entry 3),[^103^, ^131^] it is possible to obtain four SLO with ‘R’ group variations, starting with diverse monomers *a, b*, and *c types* (see Scheme 2). Syringyl‐type (S) homopolymer (SLO 4b) and p‐hydroxyphenyl‐type (H) homopolymer (SLO 4c) were obtained from monomers b and c. Guaiacyl/syringyl‐type (G/S) heteropolymer (SLO 2d) synthesized from a 1 : 1 (mol/mol) mixture of monomers *a* and *b*. Structures are analyzed by NMR and MALDI‐TOF‐MS (Table 2, Entry 10).

Computational simulations have investigated the formation of β‐5 type dimers through radical dimerization (Entry 4), revealing that the trans relationship of the substituents is the most stable configuration.^[226]^ β‐5 Type B, C, and D can be synthesized using various methods to obtain distinct ‘R’ groups. For instance, β‐5‐Type B can be obtained through the radical dimerization of isoeugenol, as detailed by Chen et al.^[227]^ as well as enzymatic reactions by Graham et al.^[224]^ offering good yields without complex mechanisms. These models can be further modified to incorporate ester groups (β‐5 Type C) or hydroxyl groups (β‐5 Type D) to enhance their structures. The chemical and enzymatic dimerization of ferrulate ester provides access to β‐5 Type C models, with the enzymatic approach showing more promise for scaling up. On the other hand, β‐5 Type D can be achieved by reducing the ester groups using LiAlH_4_ or diisobutylaluminium hydride (DIBAL‐H), or through dimerization with Ag_2_O from coniferyl alcohol. A concise, robust, and highly efficient synthesis method was successfully implemented to obtain heavy oligomers, producing a novel hexameric lignin model compound (SLO 8). This methodology showcased substantial advancements in ecological considerations and yielded auspicious outcomes concerning yield and scalability. Remarkably, the central dibromo SLO 5, which can be obtained with four steps mechanism from vanillin, demonstrated remarkable versatility as a building block, enabling the synthesis of a diverse range of hexameric and octameric lignin model compounds as Scheme 3. These newly synthesized oligomers possess a significant advantage as they incorporate three of the most predominant bond types commonly observed in natural lignin structures.^[224]^

Superior yields have been attained in the synthesis of β‐β dimers (Entry 5) by employing enzymatic methods,^[228]^ in contrast to chemical approaches. However, despite these advancements, multigram scalability, toxicity of reactive, and purification steps remain an ongoing challenge for chemical methods. In the case of syringaresinol, when employing copper (II) sulfate as a catalyst, a yield of 67 % can be obtained. Conversely, utilizing the enzymatic route with 2,6‐dimethoxy‐4‐allylphenol as the starting material allows for yields ranging from 81 % to 93 % to be achieved. This method already has kinetic parameters and purification processes, so this standard is also commercially available but at a high cost. Lancefield and Westwood (Entry 6)^[105]^ propose to obtain syringaresinol (SLO 11) from monomers in a multigram scale without any chromatographic purifications, and with two more steps, detailed in Scheme 4; it was possible to obtain hardwood (SLO 12), softwood (SLO 13), and phenolic (SLO 14) models. This syringaresinol also could be obtained by separating the glucoside ring from the *(−)‐Syringaresinol‐4‐O‐β‐D‐glucosid*, which is a commercial standard (*CAS:137038‐13‐2*).

Additionally, Karhunen et al.^[229]^ propose chemical and enzymatic methods to produce three multi‐linkage molecules (Entry 7) and the mechanisms to obtain both a monomer and a tetramer proposed by Li et al..^[230]^ (Entries 8–9), to study the hydrogenolysis of 4‐O‐5 diaryl ether units. Scheme 5 shows a pathway with 6 steps to build the 4‐O‐5 (SLO 18) that involves oxidative dimerization, benzylic bromination, methylation, Wittig reaction, reduction, and hydrogenation. Scheme 6 is the synthetic pathway for preparing 4‐O‐5 SLO 19, which includes ester formation, Lithium diisopropylamide reaction, reduction, and hydrogenation. For both structures, the presence of 4‐O‐5 linkages in lignin has been confirmed through various studies, including 2D NMR spectroscopy.

The resulting SLO can be characterized using the analytical techniques mentioned in Section 2, including thermal analysis using TGA and DSC, to determine their structure, molecular weight distribution, and chemical and thermophysical properties. Molecular simulations DFT can complement these experimental techniques by calculating spectra and predicting properties such as volume, molecular surface, and solubility. These computational and experimental results will be invaluable for using these structures as surrogate molecules in modeling equilibrium phase separation systems, such as liquid‐liquid (LLE) and vapor‐liquid (VLE) equilibria. Furthermore, they enable the performance of experimental measurements to calculate parameters currently unknown for these types of molecules, thus advancing the field of lignin research and its practical applications in various industrial processes.

By integrating these analytical and simulation methods, researchers can comprehensively understand SLO properties, facilitating their application in predictive modeling and experimental validation. This holistic approach enhances the accuracy of lignin conversion processes and provides essential data for developing new materials and chemicals derived from lignin, underscoring the importance of these synthesized model compounds in theoretical and applied research.

### 3.2 Purification techniques of SLO structures for application as standards

The purification of lignin oligomeric structures (SLO) requires the use of selective and efficient techniques to isolate high‐purity fractions that meet analytical standards (≥90 %). A compilation of experimentally tested and simulation‐validated methods is gathered in Table 8 indicating a combination of chromatographic, precipitation, crystallization, and solvent‐based techniques, each with unique advantages and scalability issues.

**Table 8:** Comparative Analysis of Purification Techniques for SLO.

Lignin Model	Technique	Observations	Ref.
β‐O‐4, β‐5, β‐β dimers β–β Model Compound: Eudesmin (SLO 10)	**CC‐ Silica gel** **Flash column chromatography** **Eluent**: ethyl acetate/hexane or acetone‐based systems Crystallization and recrystallization	**Advantages**: Effective for purifying a broad range of lignin model compounds. **Limitations**: Less efficient for high‐molecular‐weight oligomers	[25,222,223]
β‐O‐4 trimer and tetramer intermediates, as well as final structures (Scheme 1)	CC‐ silica gel **Eluents**: methanol, ethyl acetate/hexane**, ethyl acetate**	**Advantages**: **CC** effectively separated short‐chain and long‐chain oligomers **Limitations**: CC methods may not completely remove all small molecular impurities	[23,109]
**SLO 12: hardwood lignin model** containing **β‐O‐4 and β**–**β** (resinol) units	Precipitation in **acidified water** Reprecipitation in **diethyl ether (Et₂O)**.	**Advantages**: Scalable processes with batch or continuous acidification methods. **Limitations**: Acidification may cause precipitation of non‐lignin impurities, requiring further purification.	[105]
β‐5 dimers	Crystallisation directly from the crude reaction mixtures	**Advantages**: Can achieve high‐purity fractions, especially for well‐defined synthetic lignin oligomers (**Yields over 70 %)** **Limitations**: Requires large volumes of solvents, precise **temperature contr**ol, cooling, or slow eVaporation (sometimes very low temperatures.	
Intermediates shown in Scheme 3 (SLO5, SLO6)&SLO9	CC‐ Silica gel **Eluent: Ethyl acetate/n‐hexane** Recrystallization Filtration & Precipitation Solvent Extraction and Washing	**Advantages**: Combining several techniques achieves high purity levels due to better selectivity **Limitations**: CC was still necessary for some key intermediates, increasing time and solvent consumption. These are expensive processes that are difficult to scale up together.	[224,227]
Diaryl model molecules, ether dimeric, trimeric, and tetrameric (SLO 18), as well as intermediates shown in Scheme 5	Crystallization Final separation and identification by UHPLC‐QTOF‐MS	**Advantages**: Crystallization with different solvents and conditions is useful for purifying various intermediates and final products. **Limitations**: Crystallization was only applicable to specific intermediates, requiring alternative methods for some compounds (UHPLC to separate the pure peak).	[230]
Syringaresinol β–β dimer (SLO 10)	Silica Gel Flash Chromatography	**Advantages**: High yield without the need for further purification processes. **Limitations**: Flash chromatography is not always efficient for large‐scale production due to limitations in column loading capacity.	^[228]^

The biggest challenge lays in the up‐scaling of the separation process in an economically viable manner. Production, precipitation, solvent fractionation, and selective extraction offer the best balance between cost, scalability, and efficiency, enabling the production of good‐quality SLO. However, achieving the desired purity standards cannot always be guaranteed. The feasibility of the use of each of these techniques largely depends on the synthesis method and the structural characteristics of the target compound. To enhance these processes and improve their applicability to SLO, the integration of theoretical analysis and predictive models [89,235,236] – such as those for liquid‐liquid, vapor‐liquid, and solid‐liquid phase equilibria – plays a crucial role in designing and optimizing operating conditions.

For high‐purity standards for analytical applications, crystallization and column chromatography remain the most suitable techniques. However, these methods are time‐intensive, require high‐purity solvents, and are unfeasible for large‐scale production. Despite these limitations, both the pharmaceutical and cosmetic industries rely on these technologies for their high‐value products, where purity takes precedence over quantity. This aligns with the requirements for analytical standards, where ensuring substance purity is paramount, and the necessary amounts typically range from milligrams to grams. However, this approach has obvious implications for the price range of the products, possibly excluding researcher groups from smaller institutions, developing countries, or those with limited funding, ultimately limiting its overall utility and impact.

## 4 Challenges and Opportunities

### 4.1 Scientific Gaps and Challenges

It is critical to recognize that while significant progress has been made in the study of LO, much remains unknown. A pressing issue is to thoroughly understand the precise structures of LO, as our current knowledge is based on incomplete or subjective information. The characterization of LO presents several difficulties due to their heterogeneity, which can range from monomers to structures with up to 70 monomeric units. LO exhibit diversity in functional groups and similar or amphipathic chemical properties (having both polar and non‐polar parts in the same structure), greatly complicating their separation by conventional methods such as solvent extraction.^[17]^

The lack of commercially available standards for LO or similar model molecules poses significant limitations in their structural characterization. Using alternative standards, such as polystyrene in SEC/GPC, has certainly led to significant errors and inaccurate Data interpretation due to structural differences between these standards and LO. The magnitude of the error caused by such measurements is still unknown, creating a substantial scientific gap that requires addressing a viable alternative.

According to the scientific literature described in this review, several methods, including chemical and enzymatic approaches, are employed to obtain a variety of SLO, mainly dimers to octamers containing the most common bonds. This analysis helps determine which methods are most suitable for improving production and generating an adequate amount of SLO for further investigation and possible standard production.

It is worth noting that the methods described in the literature have potential for reproducibility and validation, although such validations have not yet been reported. Therefore, it is possible and recommended that these structures be produced again in the laboratory to allow a more complete characterization. This characterization should extend beyond merely confirming the presence of bonds, monomeric units, and size using NMR and GPC techniques. It should encompass a combination of various chromatographic and spectrophotometric analytical methods to provide more robust information for a deeper understanding of their nature.

Obtaining MS spectra from SLO with proper ionization is a crucial step. Soft ionization is recommended for large and complex structures where it is desired to preserve the molecular integrity and obtaining spectra that reflect intact molecular ions without extensive fragmentation. ESI is appropriate for large and polar molecules, while APPI is effective for less polar compounds and can complement ESI. It provides strong ionization efficiency for various compounds, including aromatic molecules typical of lignin derivatives. With minimal fragmentation, MALDI allows for determining molecular weights and the distribution of oligomers with high accuracy.

Due to their high reproducibility, strong ionization techniques can fully fragment the structures (destructive technique) and provide fragmentation patterns that serve as a fingerprint for compounds. EI ionization (bombarding the sample with high‐energy electrons, typically 70 eV) is generally coupled to GC and GC×GC chromatography. EI is commonly used for small to medium‐sized organic molecules up to 1250 Da, such as dimers, trimers, and some tetramers, with prior derivatization and under particular conditions (high‐temperature column and a programmable temperature vaporizer (PTV) injector).^[106]^ Considering the high temperatures required to vaporize the analytes, the thermal lability of SLO must be taken into account, as it may lead to bond breaking and prevent the observation of the original SLO mass spectrum.

CID ionization is widely used in tandem (MS/MS) for the structural analysis of biomolecules and complex organic molecules like SLO. It provides detailed structural information and can be used to study specific fragments of larger molecules. Due to the complexity of the spectra from larger structures, complex Data processing is required. As in EI, the importance of starting with the analysis of known and less complex SLO structures, such as homogeneous dimers and trimers, is highlighted in order to obtain new information and knowledge gradually. As these structures are understood, their complexity (adding heterogeneities) and size can be increased.

**Figure 7:**
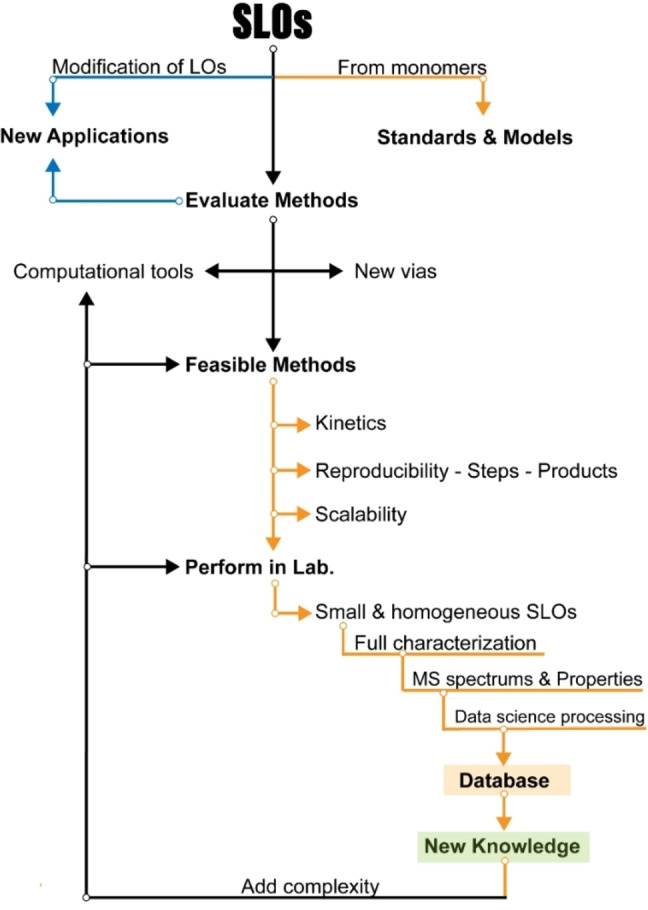
Decision paths associated with the design of SLO for new applications (blue arrows) and standards (orange arrows).

Given the significant polydispersity of LO present in lignin depolymerization products, many fully characterized SLO are also needed to serve as models and standards for identifying similar LO and establishing calibration curves for quantitative purposes whenever possible. Figure 7 summarizes the decision paths associated with the design of SLO as standards and their applications.

Until now, several cases in the literature show good results even with low to intermediate yields due to the complexity of the synthesis methods, which comprise several reaction steps (subsection 3.1.1). Moreover, some methods show potential for scaling up to multigram quantities. However, a comprehensive study of the proposed methods and the specific conditions of the chemical reactions to evaluate their reproducibility and standardization of their stages and products has not yet been conducted.

Evaluating the synthesis and purification methods requires a detailed study of the mechanisms using computational tools to determine possible improvements or simplification of steps where possible. Connie et al.[^233^, ^234^] propose methodologies for the analysis of chemical reactions using the open‐source software package RMG (Reaction Mechanism Generator)^[233]^ developed by the Green Group at MIT, to help researchers model physical processes by automatically generating mechanisms. Similarly, commercial and open‐source DFT and QSPR software can be used to analyze mechanisms and molecules, such as Gaussian, ORCA, Spartan, Q‐Chem, and others. This theoretical analysis must be accompanied by experimental validation in the laboratory to glimpse the best pathways that offer viability to become production methods for LO chemical standards.

Obtaining viable model molecules as standards is a complex and time‐consuming task due to the complete characterization required and the need to monitor their stability over time and standardize them. The substance must also have a high purity level, meaning it must be free of any contaminants that could affect analytical measurements and stability. For analytical standards, guaranteed purities of≥90 % are generally considered necessary, so having a good synthesis method is not enough; a good purification method is also required. Such a standard must have a well‐defined chemical structure and molecular weight determined using robust and reliable analytical techniques such as HPLC‐DAD/IR, GC‐FID/MS, and similar methods. Stability over time must be ensured by assessing the molecule‘s longevity under various conditions.

To scale up the production of SLO for both chemical standards (multigram quantities) and industrial applications (larger amounts), it is necessary to compare the selected methods in terms of scalability, cost‐effectiveness, and efficiency. Factors such as reaction yield, kinetics, reaction times, catalyst requirements, and post‐processing must be considered to isolate the desired products with sufficient purity. Additionally, the availability of raw materials, compatibility with equipment, and manageable operating conditions that determine the production potential must be considered when identifying the most feasible methods for large‐scale SLO production. Therefore, it is essential to study the kinetics of these chemical reaction mechanisms and their specific characteristics individually to determine the most viable ones for future scaling.

To achieve this, laboratory experiments should be conducted, combining various robust analytical techniques (as discussed in Section 2) and incorporating phenomenological modeling and computational analysis. These approaches help understand the nature of these chemical reactions and phenomena that are not easily observable during laboratory tests or through instrumentation monitoring. This comprehensive evaluation will provide a solid foundation for the efficient and scalable production of SLO, ensuring that the methods chosen are both practical and economically viable for large‐scale applications.

### 4.2 Future Directions and Opportunities

The field of synthesizing SLO and their role in understanding the depolymerization products of lignocellulosic biomass and lignin, covering the different production pathways defined in Section 3 and Section 4.1, along with the decision pathways illustrated in Figure 7, offers several promising perspectives for future research.

One of the most exciting future directions for SLO lies in exploring increasingly complex chemical structures. As our understanding of simple SLO improves and the MS spectrum database grows, researchers can gradually introduce more complexity by adding various functional groups and heterogeneities. These modifications can initially be added theoretically through DFT simulations to predict MS spectra and other properties. Subsequently, laboratory synthesis methods can be implemented, and the chemical structures can be validated. This will enable the study of more realistic models that closely resemble the LO present in the depolymerization products, ultimately aiding in developing lignin valorization processes. The ability to synthesize and analyze these complex structures will also enhance our understanding of the fundamental chemistry and reactivity of lignin itself.

Advancing SLO research and applications requires a highly interdisciplinary approach, bringing together scientists from chemistry, biochemistry, chemical engineering, materials science, and computational sciences. Collaborative efforts among these diverse fields can drive innovation in both the synthesis and application of SLO. E. g. chemists can develop new synthetic pathways and analytical methods, biochemists can explore enzymatic methods to create new SLO, material scientists can investigate the properties of SLO‐derived materials, and engineers can design separation processes, modeling, and simulation as well as data processing to feed into the database. This collaborative approach can significantly accelerate progress in research and minimize existing knowledge gaps.

Theoretical research, supported by advanced computational tools, plays a crucial role in developing viable SLO as standards and databases. Computational chemistry techniques such as DFT and Quantum Mechanics/Molecular Mechanics (QM/MM) can be used to predict the behavior and properties of SLO. These tools allow the exploration of reaction mechanisms, stability, electronic properties, and the prediction of MS, NMR, and FTIR spectra, providing valuable information that guides experimental work and Database. Integrating computational modeling with experimental validation can streamline the development of new SLO and optimize their production processes.

Data science and creating new, open‐access databases are essential for advancing SLO research. By compiling and sharing data on the properties, spectra, and synthesis methods of SLO, researchers can facilitate collaboration and accelerate discovery. Databases that include MS‐spectral data and other analytical information can serve as valuable resources for identifying unknown lignin‐derived compounds and establishing standard protocols. Open‐access platforms enable the dissemination of knowledge and resources across the scientific community, promoting transparency and reproducibility in research.

## 5 Conclusions

Despite being an abundant natural polymer, lignin is predominantly used as a source of process heat due to its complex and unreactive molecular structure, which makes it difficult to analyze and valorize through alternative routes. Technical lignin of varying qualities and contaminants is available, but using hydrolysis to break the lignocellulosic matrix and remove the sugar fraction leads to a depolymerized lignin product that behaves differently from natural lignin.

Lignin depolymerization is perhaps the most promising way to validate lignin, thereby obtaining liquid products rich in lignin oligomers, especially FPBO and BC. However, the exact pathways that lead to the breakdown of the lignin structures and the formation of these products are still not well known. Likewise, these liquid products are composed of several LO that have not yet been completely identified and characterized due to their high chemical variety and size, which makes their elucidation difficult.

The critical role of SLO in overcoming these obstacles is emphasized. SLO can not only facilitate the identification of LO present in depolymerization products to facilitate their fractionation but also improve our understanding of the mechanisms of thermochemical conversion of lignin, the development of catalysts, and the optimization of process conditions. The design and commercial production of SLO can play a fundamental role in overcoming current analytical and modeling obstacles by facilitating the identification of LO components being used as standards or model compounds, creating databases and advancing our understanding of the complex thermochemical conversion reactions involving lignin feedstocks.

Obtaining SLO that can be used as standards requires a comprehensive approach. Researchers are advised to focus on understanding and improving the reaction mechanisms and required conditions of chemical and enzymatic methods to identify the most suitable ones for industrialization and standardization. These tasks will involve integrating laboratory experiments, developing specialized methodologies with advanced analytical techniques, and conducting theoretical studies under computational analysis that allow calculating and predicting results or parameters and variables that cannot be measured experimentally. Understanding polymerization mechanisms and catalyst effects will enable the synthesis of desirable lignin‐based polymers for various applications, such as model molecules, and also potentially replace plastics and develop green materials. Collaborative efforts, interdisciplinary research, and green chemistry principles are essential for advancing the field of lignin oligomer synthesis and valorization.

Separation and purification processes and techniques are crucial to obtaining high‐purity products. Therefore, combined extraction, chromatographic, and/or membrane separation techniques are required, which can be avoided and improved using model SLO. Thinking outside the box by considering methods not yet employed in the field of lignin oligomers is surely the path to follow; one could con

## Supporting Information

Supplementary Information review SLO 1.docx: presents most SLOs in an easy‐to‐read manner and provides estimated thermophysical properties for these model molecules;

Supplementary Information review SLO 2.xlsx: contains parameters for the estimation of temperature‐dependent thermophysical properties for most SLO.

The authors have cited additional references within the Supporting Information.

## Author Contributions

**Myriam Rojas**: Conceptualization, data gathering, manuscript writing and edition, preparation of supporting information, review of manuscript, illustration, and visualization. **Frederico G. Fonseca**: Conceptualization, data gathering, manuscript writing, preparation of supporting information. Ursel Hornung: Conceptualization, data gathering, manuscript writing, Axel Funke: Conceptualization, review of manuscript with significant changes, Nicolaus Dahmen: Conceptualization, review of manuscript with changes

## Conflict of Interests

The authors declare no conflict of interest.

## Biographical Information

*Myriam Rojas holds a Ph.D. in Engineering – Energy Systems from the Department of Processes and Energy at Universidad Nacional de Colombia. She was a Guest Researcher at University of Groningen, The Netherlands. She completed her first postdoctoral research at ICIPC, Colombia, focusing on chemical recycling of plastic waste, project management, and sustainability. Her current postdoctoral research centers on synthetic lignin oligomers for analytical applications and phase equilibrium modeling at the Karlsruhe Institute of Technology ‐KIT, Germany. Myriam has extensive experience in biomass and waste processing, biobased chemical extraction and purification, thermochemical processes, and analytical techniques, with strong skills in experimental design and R&D project management*.

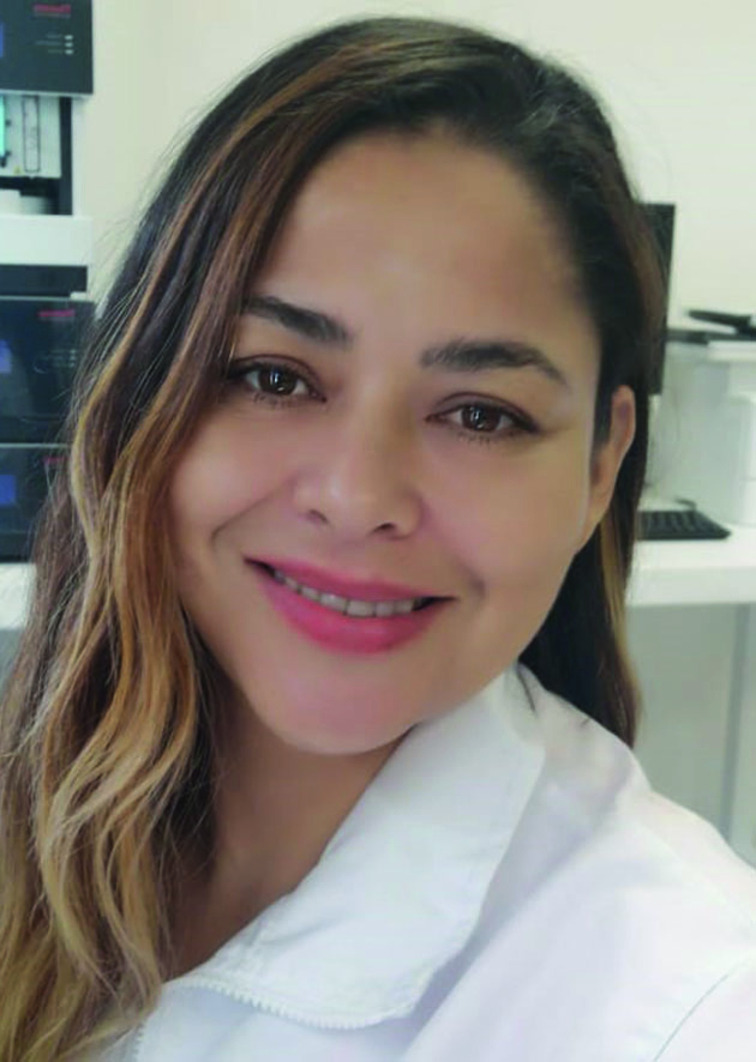

## Biographical Information

*Frederico G Fonseca is a chemical engineer passionate about sustainable energy solutions. Born in Lisbon, Portugal, he earned his BSc and MSc in Chemical Engineering at IST – University of Lisbon and completed their Ph.D. at the Karlsruhe Institute of Technology in 2023. With over a decade of active research experience, the researcher has significantly contributed to pyrolysis, liquefaction, and biofuels. His work has focused on developing innovative methods for converting biomass into sustainable energy sources while reducing greenhouse gas emissions. Currently, Frederico is exploring ways to decarbonize industrial systems, leveraging his expertise to create a more sustainable future*.

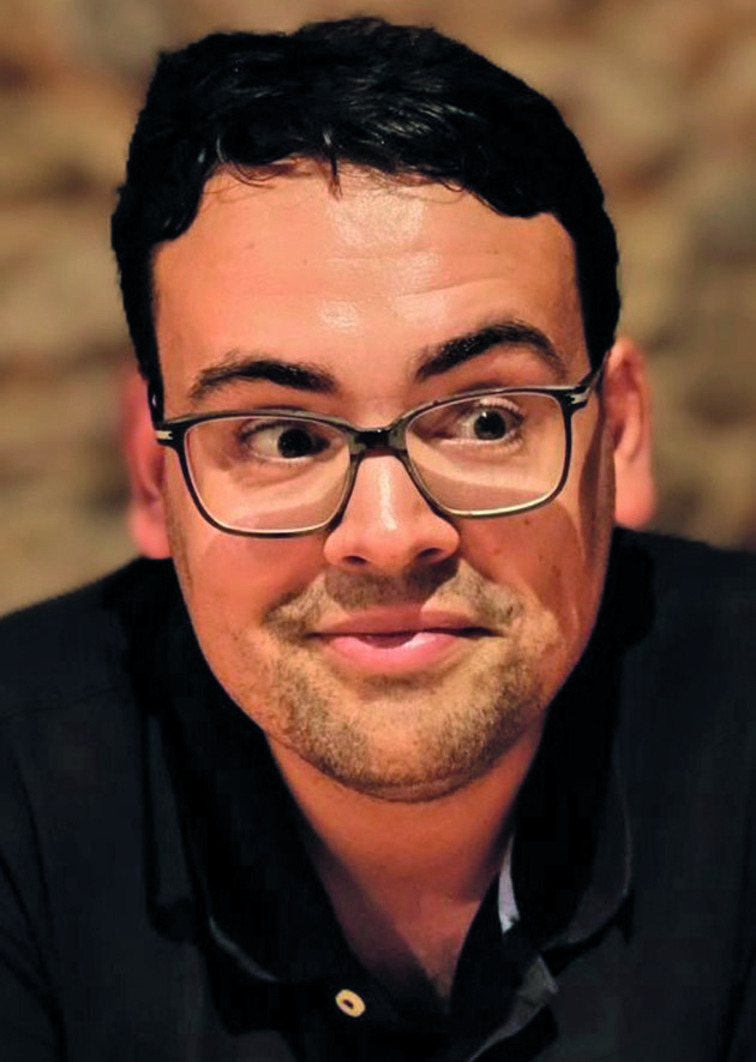

## Biographical Information

*Ursel Hornung studied chemistry at the University of Darmstadt and earned her Ph.D. from the University of Kaiserslautern on the thermal degradation of plastics. From 1996, she worked at the University of Karlsruhe on the staged pyrolysis of plastic mixtures. Since 2003, in the Karlsruhe Research Center, and performed kinetic studies on biomass gasification and coupled pyrolysis with low‐temperature reforming. Since 2010 she has been a research group leader at the IKFT‐KIT, focusingon the production of platform chemicals from biomass, especially via hydrothermal liquefaction. From 2018 to 2021, Ursel was active as a Reader at the University of Birmingham, UK*.

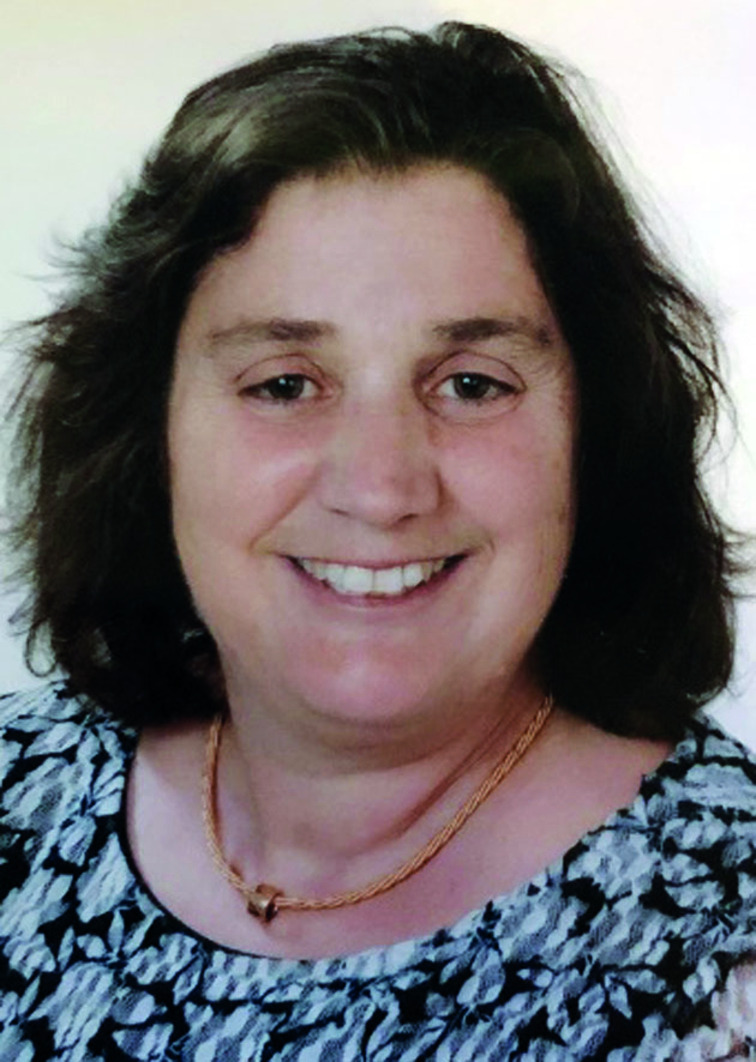

## Biographical Information

*Axel Funke studied Energy and Process Engineering at Technische Universität Berlin, Germany, and the Royal Institute of Technology, Sweden. He conducted received his Ph.D. from Technische Universität Berlin in the field of hydrothermal carbonization of waste biomass. His postdoctoral work at the Leibniz Institute in Potsdam centered on biomass conversion to biochar. Since 2013, he has led fast pyrolysis technology development at the Karlsruhe Institute of Technology – KIT, contributing to the bioliq* ^*®*^ *project for producing liquid fuels from biomass residues and engineering thermochemical biomass conversion technologies*.

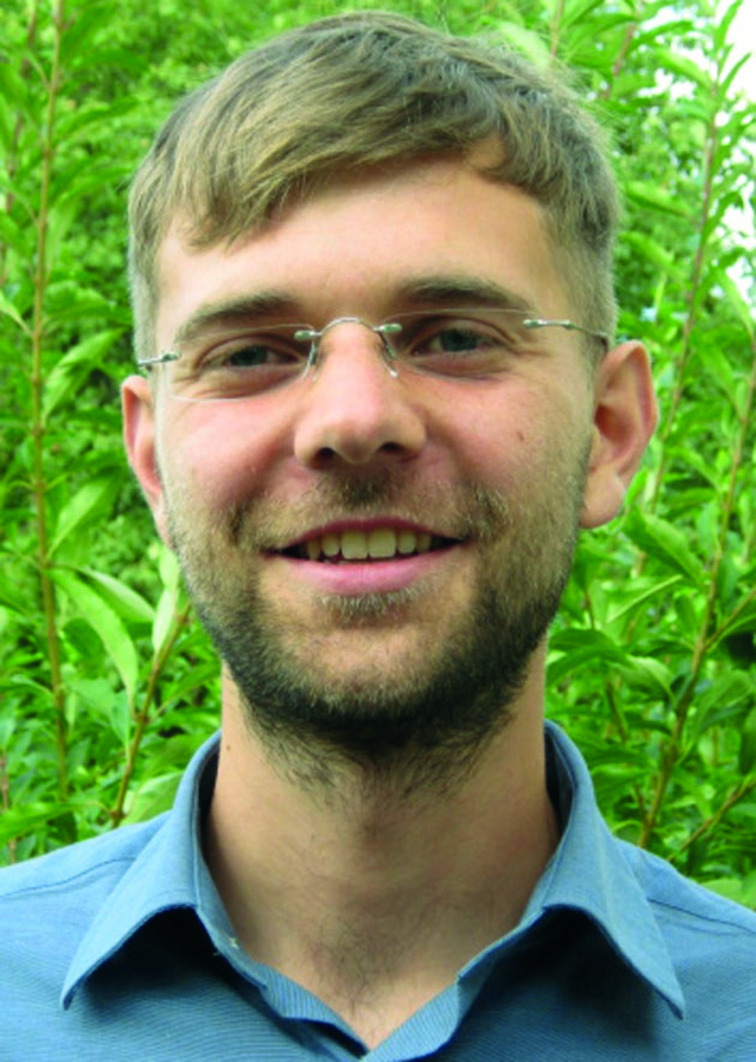

## Biographical Information

*Nicolaus Dahmen studied chemistry at the Ruhr University Bochum. After earning his Ph.D. in mixed‐phase thermodynamics in 1992, he joined the Karlsruhe Research Center (now KIT), where he established a group focused on high‐pressure processes for chemical reactions and novel separation methods. In 2000, he became head of high‐pressure process technology. By 2005, took the role of project manager of the bioliq® project. Nicolaus habilitated in process development with supercritical fluids in 2010 and became a Leading Scientist at KIT in 2014, taking the role of department leader on thermochemical biomass conversion at IKFT‐KIT, and his current scientific output specializes in renewable resource conversion*.

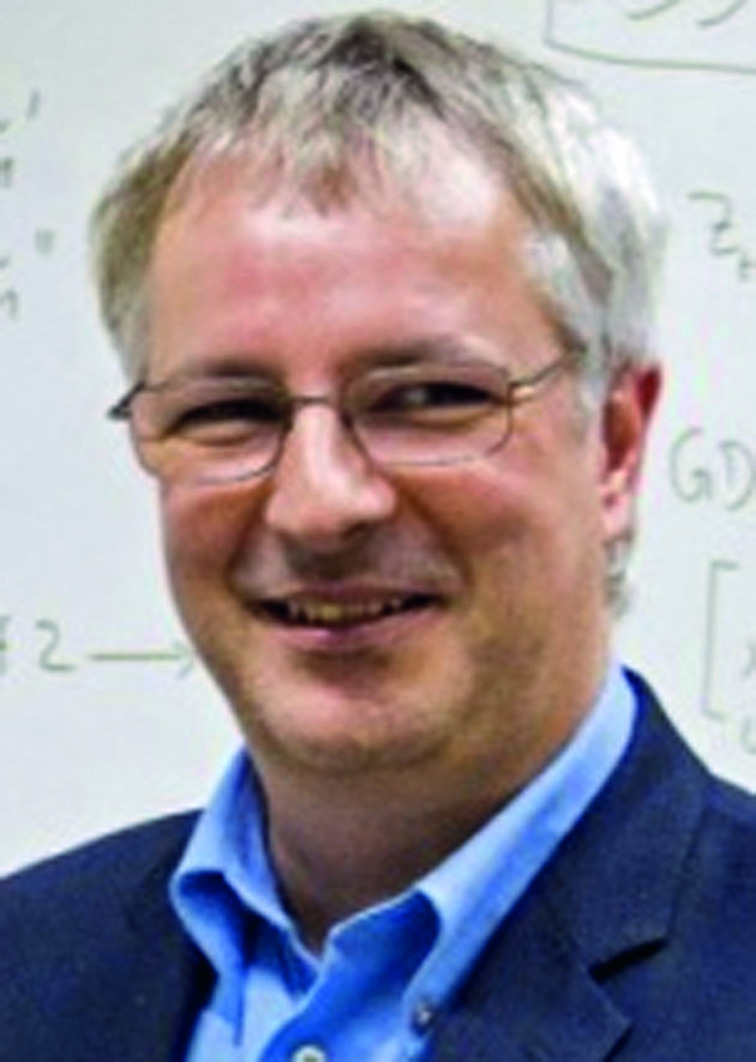

## Supporting information

As a service to our authors and readers, this journal provides supporting information supplied by the authors. Such materials are peer reviewed and may be re‐organized for online delivery, but are not copy‐edited or typeset. Technical support issues arising from supporting information (other than missing files) should be addressed to the authors.

Supporting Information

Supporting Information
