# Supplementary material for: Synthetic Lignin Oligomers: Analytical Techniques, Challenges, and Opportunities
Source: ChemSusChem. 2025 Mar 17;18(10):e202402334. doi: 10.1002/cssc.202402334 (PMC12094155; doi:10.1002/cssc.202402334)
Supplement: Supplementary file 1 — Supporting Information [file CSSC-18-e202402334-s002.pdf]

ChemSusChem

Supporting Information

Synthetic Lignin Oligomers: Analytical Techniques, Challenges, and Opportunities

Myriam Rojas,* Frederico G. Fonseca, Ursel Hornung, Axel Funke, and Nicolaus Dahmen

Supporting information I

Synthetic Lignin Oligomers: Analytical Techniques, Challenges, and Opportunities

Myriam Rojas *,[a], Frederico G. Fonseca [a,b], Ursel Hornung [a], Axel Funke [a], and Nicolaus Dahmen [a]

[a] Dr. Myriam R. (Corresponding Author), Dr. Frederico F., Dr. Ursel H., Dr. Axel F., Prof. Nicolaus D.
Department: Scale-up of processes with renewable carbon sources
Institution: Institute of Catalysis Research and Technology – Karlsruhe Institute of Technology (IKFT-KIT)
Address: Hermann-von-Helmholtz-Platz 1, 76344 Eggenstein-Leopoldshafen
E-mail: myriam.salas@kit.edu

[b] Dr. Frederico G. Fonseca
Department: Simulation and Virtual Design
Institution: Institute for Low-Carbon Industrial Processes – German Aerospace Agency (DLR)
Address: Walther-Pauer-Straße 5, 03046 Cottbus

Chemical structures of SLO, Molecular weight and m/z values.

Dimers

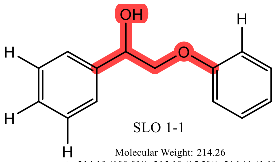

SLO 1-1
Molecular Weight: 214.26
m/z: 214.10 (100.0%), 215.10 (15.2%), 216.11 (1.1%)

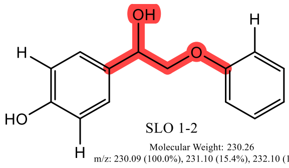

SLO 1-2
Molecular Weight: 230.26
m/z: 230.09 (100.0%), 231.10 (15.4%), 232.10 (1.7%)

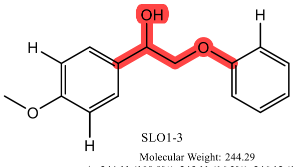

SLO1-3
Molecular Weight: 244.29
m/z: 244.11 (100.0%), 245.11 (16.3%), 246.12 (1.3%)

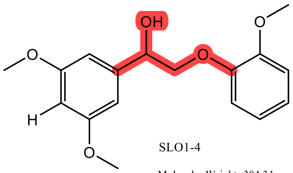

SLO1-4
Molecular Weight: 304.34
m/z: 304.13 (100.0%), 305.13 (18.4%), 306.14 (2.7%)

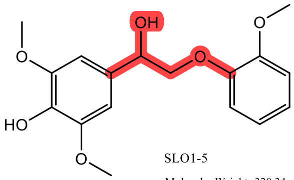

SLO1-5
Molecular Weight: 320.34
m/z: 320.13 (100.0%), 321.13 (18.8%), 322.13 (2.9%)

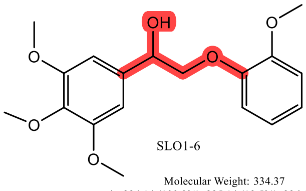

SLO1-6
Molecular Weight: 334.37
m/z: 334.14 (100.0%), 335.14 (19.5%), 336.15 (3.1%)

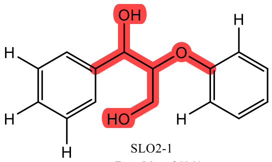

SLO2-1
Exact Mass: 244.11
Molecular Weight: 244.29

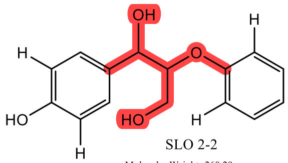

SLO 2-2
Molecular Weight: 260.29
m/z: 260.10 (100.0%), 261.11 (16.6%), 262.11 (2.1%)

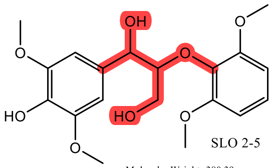

SLO 2-5

Molecular Weight: 380.39
m/z: 380.15 (100.0%), 381.15 (21.1%), 382.15 (3.7%)

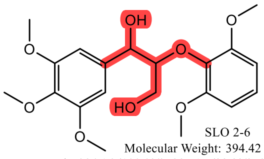

SLO 2-6
Molecular Weight: 394.42
m/z: 394.16 (100.0%), 395.17 (22.2%), 396.17 (4.0%)

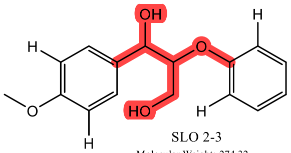

SLO 2-3
Molecular Weight: 274.32
m/z: 274.12 (100.0%), 275.12 (17.5%), 276.13 (1.5%)

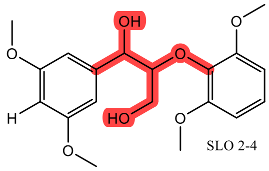

SLO 2-4

Molecular Weight: 364.39
m/z: 364.15 (100.0%), 365.16 (21.1%), 366.16 (3.6%)

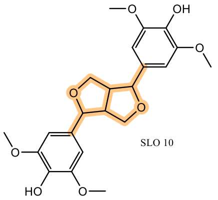

SLO 10

Molecular Weight: 418.44
m/z: 418.16 (100.0%), 419.17 (24.4%), 420.17 (4.5%)

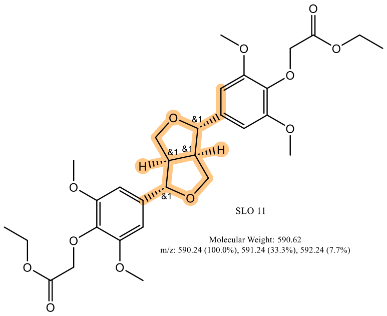

SLO 11

Molecular Weight: 590.62
m/z: 590.24 (100.0%), 591.24 (33.3%), 592.24 (7.7%)

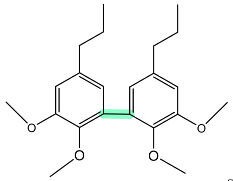

SLO 17-1 Molecular Weight: 358.48
m/z: 358.21 (100.0%), 359.22 (24.3%), 360.22 (3.6%)

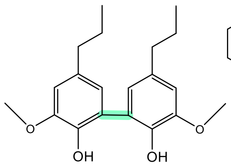

SLO 17-3 Molecular Weight: 330.42
m/z: 330.18 (100.0%), 331.19 (22.1%), 332.19 (3.1%)

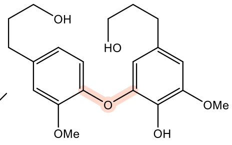

SLO 18
Molecular Weight: 362.42
m/z: 362.17 (100.0%), 363.18 (22.2%), 364.18 (3.6%)

Trimers

SLO 3 Molecular Weight: 590.62
m/z: 590.24 (100.0%), 591.24 (33.3%), 592.24 (7.7%)

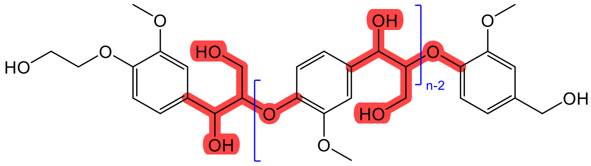

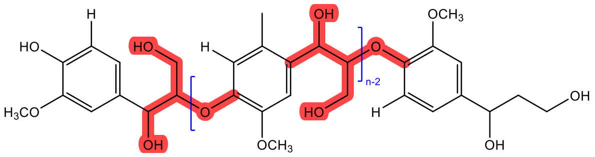

SLO 4a
Molecular Weight: 604.65
m/z: 604.25 (100.0%), 605.26 (34.4%), 606.26 (8.2%), 607.26 (1.5%)

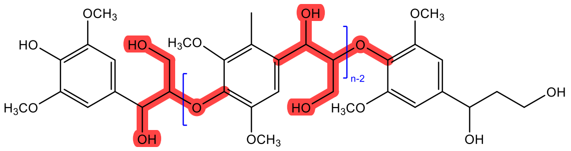

SLO 4b
Molecular Weight: 694.73
m/z: 694.28 (100.0%), 695.29 (37.9%), 696.29 (10.1%), 697.29 (2.0%)

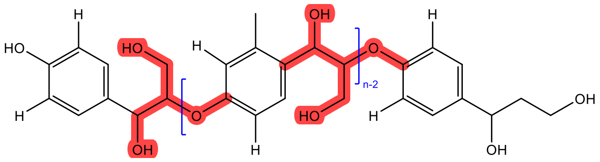

SLO 4c Molecular Weight: 514.57
m/z: 514.22 (100.0%), 515.22 (30.6%), 516.23 (4.6%), 516.22 (1.8%), 517.23 (1.0%)

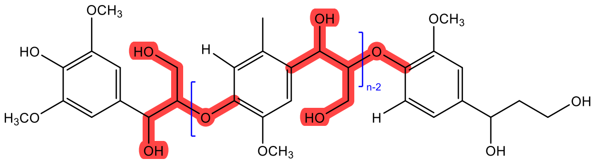

SLO 4d Exact Mass: 634.26
m/z: 634.26 (100.0%), 635.27 (35.6%), 636.27 (8.8%), 637.27 (1.6%)

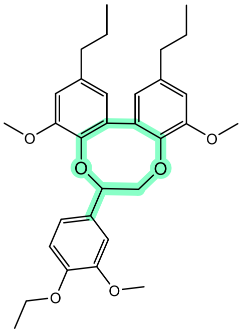

SLO 15 Molecular Weight: 506.64
m/z: 506.27 (100.0%), 507.27 (34.2%), 508.27 (6.7%)

Tetramers

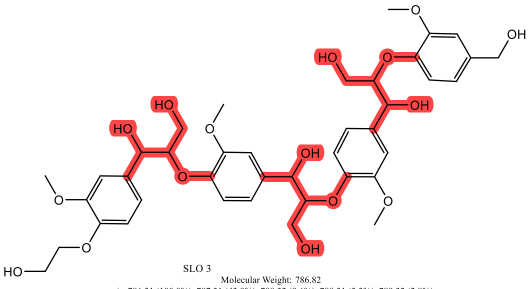

SLO 3
Molecular Weight: 786.82
m/z: 786.31 (100.0%), 787.31 (43.9%), 788.32 (9.6%), 788.31 (3.3%), 789.32 (2.8%)

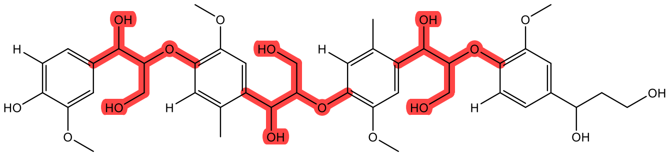

SLO 4a Molecular Weight: 814.88
m/z: 814.34 (100.0%), 815.34 (45.4%), 816.35 (13.9%), 817.35 (3.1%), 815.35 (1.2%)

SLO 4b Molecular Weight: 934.98
m/z: 934.38 (100.0%), 935.39 (51.2%), 936.39 (17.0%), 937.39 (4.1%)

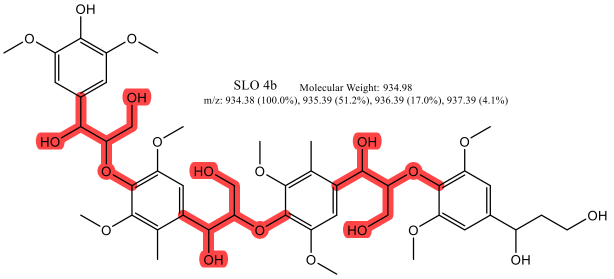

SLO 4c

Molecular Weight: 694.77

m/z: 694.30 (100.0%), 695.30 (41.6%), 696.31 (8.6%), 696.30 (2.5%), 697.31 (2.2%)

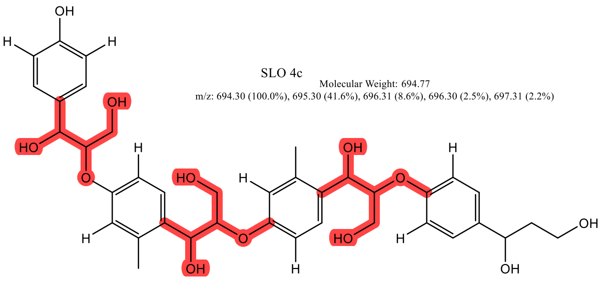

SLO 4c

Molecular Weight: 694.77

m/z: 694.30 (100.0%), 695.30 (41.6%), 696.31 (8.6%), 696.30 (2.5%), 697.31 (2.2%)

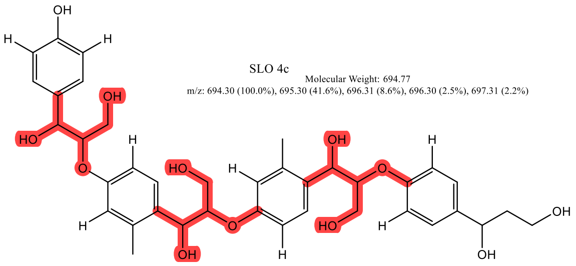

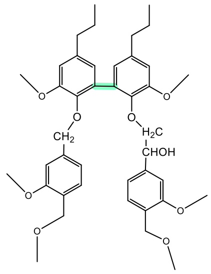

SLO 16 Molecular Weight: 688.86

m/z: 688.36 (100.0%), 689.36 (44.3%), 690.37 (11.9%), 691.37 (2.3%)

SLO 17-2 Molecular Weight: 510.67

m/z: 510.28 (100.0%), 511.28 (37.4%), 512.28 (7.4%)

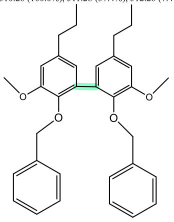

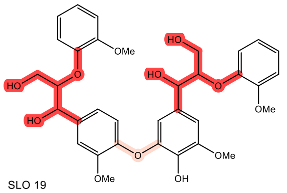

SLO 19

m/z: 638.24 (100.0%), 639.24 (37.7%), 640.24 (9.2%)

Bigger structures

SLO 9

Molecular Weight: 1393.58

m/z: 1392.61 (100.0%), 1393.61 (87.3%), 1394.61 (40.6%), 1395.62 (14.6%), 1396.62 (4.0%), 1394.62 (1.6%)

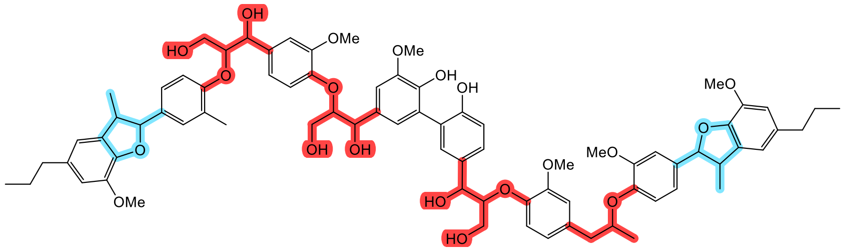

SLO 13

Molecular Weight: 1517.59

m/z: 1516.57 (100.0%), 1517.58 (88.7%), 1518.58 (44.8%), 1519.58 (16.1%), 1520.59 (2.4%), 1520.58 (2.4%), 1521.59 (1.1%)

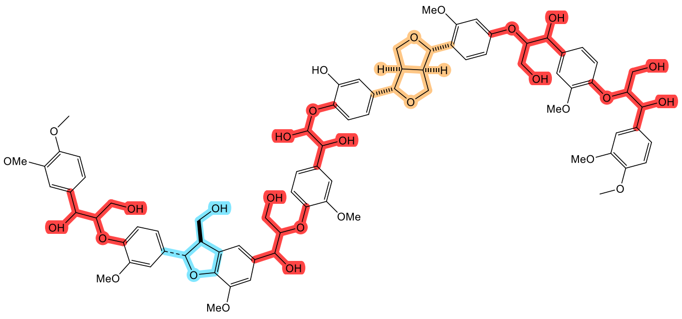

SLO 14

Molecular Weight: 1443.51

m/z: 1442.54 (100.0%), 1443.54 (85.3%), 1444.54 (40.6%), 1445.55 (10.0%), 1445.54 (4.7%), 1446.55 (4.0%), 1447.55 (1.0%)

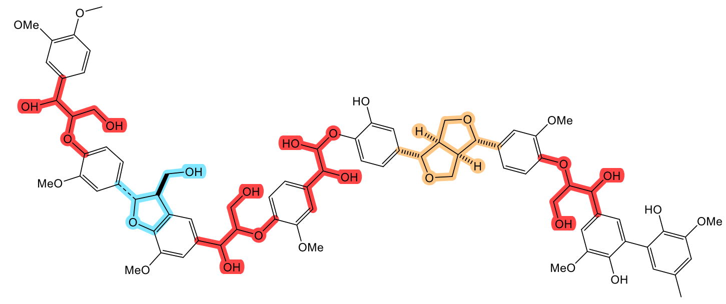

Table 1: Thermophysical parameter values estimated for the different SLOs. Methods chosen based on the work of Fonseca and Funke (2024)[1], for the case when reasonable values could be found for all structures. Formulas and parameters for the estimation of the temperature-dependent properties are provided in a separate Excel file.

ID	Formula	Molecular Weight	Normal Boiling Point	Critical Temperature	Critical Pressure	Critical Volume	Ideal Gas Heat Capacity*	Standard Enthalpy of Formation*	Vapor pressure*	Enthalpy of Vaporization*	Standard Liquid Volume*
Unit		g·mol^{-1}	°C	°C	bar	cm^3·mol^{-1}	J·mol^{-1}·K^{-1}	kJ·mol^{-1}	kPa	kJ·mol^{-1}	cm^3·mol^{-1}
Method			Joback[2]	Gani[3]	Nanoollal-Rarey[4]	Joback[2]	Benson[5]	Benson[5]	Nanoollal-Rarey[6]	Ducros[7–9]	ACD/Labs**
SLO1-1	$C_{14}H_{14}O_2$	214.26	414.1	496.7	30.75	634.5	235.1	-159.9	1.34×10^{-2}	128.2	188.8
SLO 1-2	$C_{14}H_{14}O_3$	230.26	494.7	546.4	39.34	600.5	255.9	-336.1	8.97×10^{-4}	120.7	184.3
SLO1-3	$C_{15}H_{16}O_3$	244.29	464.4	525.4	28.57	708.5	272.6	-314.4	1.39×10^{-3}	98.7	212.9
SLO 1-4	$C_{17}H_{20}O_5$	304.34	564.9	572.0	24.58	856.5	368.8	-621.0	1.70×10^{-5}	117.3	260.9
SLO1-5	$C_{17}H_{20}O_6$	320.34	645.6	625.9	30.56	822.5	389.6	-797.2	5.35×10^{-7}	130.4	259.3
SLO 1-6	$C_{18}H_{22}O_6$	334.37	615.2	591.4	22.89	930.5	411.8	-775.5	1.81×10^{-6}	119.7	284.9
SLO2-1	$C_{15}H_{16}O_3$	244.29	528.7	534.4	32.34	703.5	273.1	-348.7	2.59×10^{-4}	127.4	203.2
SLO 2-2	$C_{15}H_{16}O_4$	260.29	578.3	575.8	41.96	637.5	293.9	-524.9	1.69×10^{-5}	162.4	201.7
SLO2-3	$C_{15}H_{18}O_4$	274.32	548.0	528.9	30.12	745.5	316.1	-503.2	6.49×10^{-5}	125.6	227.2
SLO 2-4	$C_{19}H_{24}O_7$	364.39	729.8	614.7	24.05	999.5	445.1	-966.7	2.36×10^{-8}	155.4	299.2
SLO2-5	$C_{19}H_{24}O_8$	380.39	810.5	642.4	28.24	965.5	407.9	-764.5	1.19×10^{-9}	144.6	297.7
SLO 2-6	$C_{20}H_{26}O_8$	394.42	780.1	613.5	22.44	1073.5	488.1	-1121.2	2.08×10^{-8}	162.4	323.2
SLO3	$C_{40}H_{50}O_{16}$	786.82	1902.2	761.2	16.13	2103.5	937.6	-2351.9	6.39×10^{-27}	409.7	578.8
SLO 4-1(a)	$C_{42}H_{54}O_{16}$	814.88	2010.7	755.7	14.60	2157.5	950.8	-2180.3	2.24×10-^{27}	410.0	603.0
SLO4-2 (b)	$C_{46}H_{62}O_{20}$	934.98	2235.4	790.9	11.89	2518.5	1243.4	-3209.0	6.61×10^{-33}	481.3	699.0
SLO 4-3(c)	$C_{38}H_{46}O_{12}$	694.77	1809.6	740.8	18.71	1861.5	785.3	-1808.4	5.00×10-^{23}	405.6	507.0
SLO9	$C_{79}H_{92}O_{22}$	1393.58	3205.0	838.3	4.45	3774.5	1659.4	-3113.0	5.40×10^{-41}	635.9	1074.5
SLO 10	$C_{22}H_{26}O_8$	418.44	820.4	656.8	26.47	501.5	467.2	-1075.1	1.06×10^{-8}	183.6	326.2
SLO11	$C_{30}H_{38}O_{12}$	590.62	1166.1	708.7	12.97	1547.5	688.9	-1695.4	1.49×10^{-11}	212.9	483.4
SLO 13	$C_{77}H_{86}O_{30}$	1491.51	3694.2	897.4	7.94	3750.5	1653.9	-4024.7	2.72×10-^{58}	777.8	1052.3
SLO14	$C_{75}H_{82}O_{27}$	1415.45	3407.4	898.8	8.56	3476.5	1545.8	-3513.9	4.75×10^{-51}	681.5	1002.2
SLO 15	$C_{31}H_{38}O_6$	506.64	910.0	684.9	10.21	1510.5	648.9	-788.0	8.61×10^{-9}	161.2	458.9
SLO16	$C_{41}H_{52}O_9$	688.86	1269.1	738.9	7.09	2000.5	882.6	-1292.0	8.26×10^{-15}	253.1	614.5
SLO 17-1	$C_{22}H_{30}O_4$	358.48	602.5	619.8	14.34	1123.5	496.7	-538.6	6.56×10^{-6}	123.2	349.2
SLO17-2	$C_{34}H_{38}O_4$	510.67	930.5	690.2	9.60	1579.5	596.8	87.1	2.15×10^{-8}	158.8	470.7
SLO 17-3	$C_{20}H_{26}O_4$	330.42	663.2	644.5	22.76	907.5	401.1	-439.0	5.26×10^{-7}	140.3	298.2
SLO18	$C_{20}H_{26}O_6$	362.42	789.4	652.3	17.95	997.5	411.5	-687.6	2.85×10^{-9}	168.1	300.1
SLO 9	$C_{34}H_{38}O_{12}$	638.67	1399.5	731.7	16.80	1539.5	761.0	-1605.5	9.71×10-^{17}	301.1	482.1

*: At standard (NTP) conditions.

**: Estimated using the ACD/Labs Percepta Platform, available on the ChemSketch software package (free version).

References

(1) Fonseca, F. G. ,; Funke, A. ,. Modeling of Liquid-Vapor Phase Equilibria of Pyrolysis Bio-Oils. . *[Manuscript submitted for publication].*

(2) Joback, K. G.; Reid, R. C. Estimation of Pure-Component Properties from Group-Contributions. *Chem Eng Commun* **1987**, *57* (1–6), 233–243. https://doi.org/10.1080/00986448708960487.

(3) Constantinou, L.; Gani, R. New Group Contribution Method for Estimating Properties of Pure Compounds. *AIChE Journal* **1994**, *40* (10), 1697–1710. https://doi.org/10.1002/aic.690401011.

(4) Nannoolal, Y.; Rarey, J.; Ramjugernath, D. Estimation of Pure Component Properties: Part 2. Estimation of Critical Property Data by Group Contribution. *Fluid Phase Equilib* **2007**, *252* (1–2), 1–27. https://doi.org/10.1016/j.fluid.2006.11.014.

(5) Benson, S. W. New Methods for Estimating the Heats of Formation, Heat Capacities, and Entropies of Liquids and Gases. *J Phys Chem A* **1999**, *103* (51), 11481–11485. https://doi.org/10.1021/jp992971a.

(6) Moller, B.; Rarey, J.; Ramjugernath, D. Estimation of the Vapour Pressure of Non-Electrolyte Organic Compounds via Group Contributions and Group Interactions. *J Mol Liq* **2008**, *143* (1), 52–63. https://doi.org/10.1016/j.molliq.2008.04.020.

(7) Ducros, M.; Gruson, J. F.; Sannier, H. Estimation Des Enthalpies de Vaporisation Des Composes Organiques Liquides. Partie 1. Applications Aux Alcanes, Cycloalcanes, Alcenes, Hydrocarbures Benzeniques, Alcools, Alcanes Thiols, Chloro et Bromoalcanes, Nitriles, Esters, Acides et Aldehydes. *Thermochim Acta* **1980**, *36* (1), 39–65. https://doi.org/10.1016/0040-6031(80)80109-2.

(8) Ducros, M.; Gruson, J. F.; Sannier, H.; Velasco, I. Estimation Des Enthalpies de Vaporisation Des Composes Organiques Liquides. Partie 2. Applications Aux Ethersoxydes, Thioalcanes, Cetones et Amines. *Thermochim Acta* **1981**, *44* (2), 131–140. https://doi.org/10.1016/0040-6031(81)80035-4.

(9) Ducros, M.; Sannier, H. Estimation Des Enthalpies de Vaporisation Des Composes Organiques Liquides, Partie 3. Application Aux Hydrocarbures Insatures. *Thermochim Acta* **1982**, *54* (1–2), 153–157. https://doi.org/10.1016/0040-6031(82)85074-0.
